# A mathematical characterization of minimally sufficient robot brains

**DOI:** 10.1177/02783649231198898

**Published:** 2023-09-19

**Authors:** Basak Sakcak, Kalle G Timperi, Vadim Weinstein, Steven M LaValle

**Affiliations:** Center for Ubiquitous Computing, Faculty of Information Technology and Electrical Engineering, 6370 University of Oulu, Oulu, Finland

**Keywords:** Planning, transition systems, sensing uncertainty, sensor fusion, filtering, information spaces, machine learning, control theory, theoretical foundations

## Abstract

This paper addresses the lower limits of encoding and processing the information acquired through interactions between an internal system (robot algorithms or software) and an external system (robot body and its environment) in terms of action and observation histories. Both are modeled as transition systems. We want to know the weakest internal system that is sufficient for achieving passive (filtering) and active (planning) tasks. We introduce the notion of an *information transition system* (ITS) for the internal system which is a transition system over a space of information states that reflect a robot’s or other observer’s perspective based on limited sensing, memory, computation, and actuation. An ITS is viewed as a filter and a policy or plan is viewed as a function that labels the states of this ITS. Regardless of whether internal systems are obtained by learning algorithms, planning algorithms, or human insight, we want to know the limits of feasibility for given robot hardware and tasks. We establish, in a general setting, that minimal information transition systems (ITSs) exist up to reasonable equivalence assumptions, and are unique under some general conditions. We then apply the theory to generate new insights into several problems, including optimal sensor fusion/filtering, solving basic planning tasks, and finding minimal representations for modeling a system given input-output relations.

## 1. Introduction

Accomplishing a robot’s tasks may involve designing or employing a combination of different parts: planning algorithms, sensor fusion or filtering methods, machine learning algorithms, and control laws. Given a problem expressed in terms of a well-defined task structure, the relationship between these different parts with each other, and their relation with the task is often ignored. Each part is developed rather in isolation, heavily motivated by the long lasting traditions in robotics. For example, navigating a mobile robot to a goal configuration is typically achieved by estimating the robot configuration in a known (or an unknown) map and applying a feedback policy that relies on the estimated configuration. Indeed, for some problems it may be possible that a simpler filtering approach that does not require estimating the full state could as well be sufficient to achieve the given task. This may lead many to believe that robotics itself does not have its own, unique theoretical core (on this, we agree with (Koditschek, 2021)) and it appears as an application area for other fields; designing and testing machine learning algorithms, planning algorithms, sensor fusion methods, control laws, and so on. In our quest towards a theory that is unique to robotics and that plays a similar role to Turing machines for computer science, or $\dot{x}=f\left(x,u\right)$ over differentiable manifolds for control theory, we want to establish the limits of the intertwined notions of sensing, learning, filtering, and planning with respect to a given problem. We would like to have a general framework that allows researchers to formulate and potentially answer questions such as: Does a solution even exist to a given problem? What are the minimal necessary components to solve it? What should the best learning approach imaginable produce as a representation? Such questions would be analogous to existence and uniqueness in control and dynamical systems, or decidability and complexity (especially Kolmogorov) in theoretical computer science.

**Figure 1.**
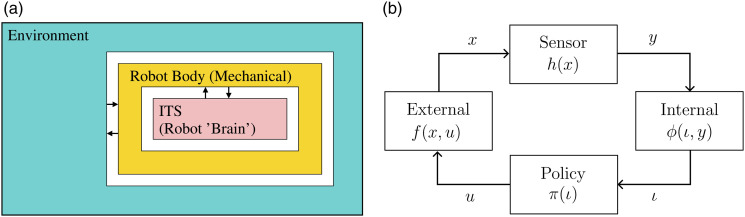
(a) The *internal* robot brain is defined as an ITS that interacts with the *external* world (robot body and environment). (b) Coupled internal and external systems mathematically capture sensing, actuation, internal computation, and the external world.

This paper proposes a mathematical robotics theory that is built from the input-output relationships between two (or more) coupled dynamical systems. For a programmable mechanical system (robot) embedded in an environment, the input-output relationships correspond to sensing and actuation between two coupled systems; an *internal system* (robot brain) and an *external system* (robot body and the environment). This relation is shown in Figure 1(a)–(b). We assume that the robot hardware is fixed, which means fixing the sensors and the actuators, and we focus on determining which necessary and sufficient conditions the internal system has to maintain for a task to be accomplished. In light of these conditions, we try to find a *minimal sufficient* internal system which corresponds to the weakest possible representation of the acquired information through interactions; reducing the internal system any further makes the problem unsolvable.

At the core of our framework is the notion of an ITS which builds on the well-studied notion of transition systems. The *information* part of an ITS comes from *information spaces* (LaValle, 2006: Chapter 11) which is developed as a foundation of planning with imperfect state information due to sensing uncertainty. The concept of *sufficient information mappings* appears therein. It is generalized in this paper, and the state space of each ITS will in fact be an information space. An internal system will be modeled as an ITS and its sufficiency and minimality with respect to a problem will be analyzed using this framework.

We categorize tasks into two classes: active and passive. Informally, a passive task corresponds to filtering and an active one corresponds to planning or control. In our work an ITS can be seen as a filter and together with its underlying information space serves as a domain over which a plan or a policy can be expressed and analyzed. We will consider a variety of information spaces, which also encompass robot configuration spaces or phase spaces, that are typically used for planning tasks. A distinction between *model-based* and *model-free* formulations will be considered too, in line with the choices commonly found in machine learning. We characterize the problems corresponding to the active and passive tasks and define notions of feasibility and minimality for *information transition systems* (ITSs) that solve these problems. In our approach to finding minimal sufficient ITSs, we will analyze the limits of reducing or collapsing the information spaces, until the lower limits of task feasibility are reached.

### 1.1. Previous work

Many of the concepts in this paper build upon (Weinstein et al., 2022), in which we recently proposed an enactivist-oriented model of cognition based on information spaces. By *enactivist* (Hutto and Myin, 2012), it is meant that the necessary brain structures emerge from sensorimotor interaction, and do not necessarily have predetermined, meaningful components as in the classical representationalist paradigm (Newell and Simon, 1972). Despite introducing a general framework, the focus of (Weinstein et al., 2022) was on emerging equivalence classes of the environment states through sensorimotor interactions. In some sense, this corresponds to finding a (minimal) sufficient sensor or a representation for the external system. In this paper, we focus on the internal system which, in turn, refers to finding a minimal filter and/or a policy, given an appropriate task description.

Most approaches in filtering can be categorized into two classes: probabilistic and combinatorial. Probabilistic filters typically rely on Bayes’ rule to propagate the obtained information (Särkkä, 2013; Thrun et al., 2005). They have been extensively used in robotics; especially for state estimation, mapping, and localization (Dissanayake et al., 2001; Zhen et al., 2017). Combinatorial filters (Kristek and Shell, 2012; Tovar et al., 2008) do not rely on probabilistic models but instead make use of nondeterministic (possibilistic) ones. The notion of information spaces (LaValle, 2006: Chapter 11) provides a general formalization that encompasses both probabilistic and combinatorial filters. In our framework based on transition systems, the set of states of a transition system will indeed be an information space. Using the notion of information spaces, several works attempted to characterize different sensors defined over the same state space and compare their power in terms of gathered information (LaValle, 2012; O’Kane and LaValle, 2008; Zhang and Shell 2021). Despite providing an elegant and exact way of solving filtering problems, the space requirements for a combinatorial filter can be high. Considering a notion of minimality, some authors addressed algorithmic reduction of filters (O’Kane and Shell, 2017; Rahmani and O’Kane, 2021; Song and O’Kane, 2012). Reducing combinatorial filters have roots in the theory of computation, where decomposition (Hartmanis, 1960; Hartmanis and Stearns, 1964) and minimization of finite automata (Moore and Mealy machines) has been a topic of active research since the 1950s.

A gap still remains between analyzing the requirements of filters or sensors for pure inference (passive filtering) and the ones needed for active tasks (planning and control) such that an *information-feedback* policy can be described. Various representations were used in the literature as a domain to define the policy. For most robotic planning problems, the domain of the policy or a plan is fixed; which is the robot configuration or the phase space (Majumdar and Tedrake, 2017; Zhu and Alonso-Mora, 2019). This corresponds to the assumption that the robot state can be fully observed or estimated with high accuracy. For problems that the state is not fully observable, *partially observable Markov decision processes* (POMDPs) (Kaelbling et al., 1998; Ross et al., 2008) and *belief spaces* (Vitus and Tomlin, 2011; Agha-Mohammadi et al., 2014) have been considered for planning. Note that POMDP literature is mostly restricted to finite state and action spaces. There is a limited literature that studied the information requirements for active tasks which corresponds to determining the weakest notion of sensing or filtering that is sufficient to accomplish a task. A notable early work showed, especially for manipulation, that one can achieve certain tasks even in the absence of sensory observations (Erdmann and Mason, 1988). In (Zhang and Shell, 2020) the authors characterize all possible sensor abstractions that are sufficient to solve a planning problem. Minimality has been addressed for specific problems regarding mobile robot navigation in (Blum and Kozen, 1978; Tovar et al., 2007). Closely related to our work, a language-theoretic formulation appears in (Saberifar et al., 2019), in which, Procrustean-graphs (p-graphs) were proposed as an abstraction to reason about interactions between a robot and its environment.

Obtaining a model from input–output relations that represents the underlying system has been a common interest for many fields ranging from control theory, machine learning, and robotics. Different approaches to this problem in the context of finite state automata were reviewed by (Pitt, 1989). In diversity-based inference (DBI) for an input–output machine (Bainbridge, 1977; Rivest and Schapire, 1993, 1994), a model of the underlying system is constructed in terms of equivalence classes of *tests* which consist of one or more consecutive actions and observations. Its probabilistic counterpart, predictive state representation (PSR) (Boots et al., 2011; Littman and Sutton, 2001), addresses the analogous problem by considering (linear) combinations of prediction vectors, which represent probabilities for test success/failure. Other than relying solely on input–output relations, a parametric model (or a class of them) can be provided for learning a representation for the underlying system (Brunnbauer et al., 2022). We will also consider this distinction between model-based and model-free within our ITS framework.

### 1.2. Contributions

The main contribution of this paper is a novel mathematical framework for analyzing and distinguishing the interactions that emerge from a robotic system embedded in an environment. We introduce the notion of ITSs as a general way to characterize the internal system (“brain”) of the robot. We then proceed to establish conditions for sufficiency and the existence of unique minimal ITSs in a very general setting. Intuitively, we establish how small the robot brain could possibly be for given goals or tasks. Anything less results in impossibility. The framework addresses both filtering and sensor fusion problems, which are passive in the sense of no controls are applied, and planning or control problems, which are active. We illustrate the scope of the framework by applying it to several problems that shed light on relationships to many existing concepts, including Kalman filters, predictive state representations, combinatorial filters, and planning over reduced information spaces.

Many of the concepts in this paper build upon (LaValle, 2006: Chapter 11; Weinstein et al., 2022). The contributions of the present paper with respect to these works are listed in the following:

  • The framework based on transition systems introduced in (Weinstein et al., 2022) is adapted into a robotic setting. We define the notions of internal and external systems within this context, together with concrete examples. Moreover, we formulate the disturbances affecting the external system model and the sensor mapping, within this framework.
  • We formally define the notion of task description (distinguishing between finite and infinitary tasks, as well as between active and passive tasks), and filtering and planning problems.
  • The notion of minimality for a transition system describing the internal system under a planning (control) task is new.
  • The ITSs corresponding to some of the information spaces presented in (LaValle, 2006: Chapter 11), which were based on intuitions, are shown to be minimal applying the proposed framework.
  • We also formalize the model-based and the model-free information spaces using the notion of coupled internal-external systems.

This paper is an expanded version of (Sakcak et al., 2022).

### 1.3. Paper structure

The remaining of the paper is organized as follows. Section 2 provides a general mathematical formulation of robot-environment interaction as transition systems. We also introduce a notion of couplings which encode various types of interactions between an internal and an external system. Section 3 then develops central notions of sufficiency and minimality over the space of possible ITSs. Section 4 applies the general concepts to address what it means to solve both passive (filtering) and active (planning/control) tasks minimally. Canonical problem families are presented that aim to capture typical problem settings in filtering and planning, from the perspective of minimal sufficient solutions. Section 5 illustrates how the theory can be applied using simple examples. Section 6 summarizes the contributions and identifies important directions for future work.

## 2. Mathematical models of robot-environment systems

### 2.1. Internal and external systems

In this paper, we consider a robot embedded in an environment and describe this system as two subsystems, named *internal* and *external*, connected through symmetric input-output relations. The external system describes the physical world, and the internal system describes the information processing “robot brain.” With “robot brain” we refer to a centralized computational component that processes sensor observations and actions. The interaction between the internal and external systems is shown in Figure 1(b). The input to the internal system is the information reflecting the state of the external system, obtained through observations (that is the output of the external system). The output of the internal system is a control command that in turn corresponds to the control input of the external system, and causes its state to change. In this sense, the state of the external system is similar to the use of the term *state* in control theory and the state of the internal system is similar to the use of the term in computer science.

The external system corresponds to the totality of the physical environment, including the robot body. Let *X* denote the set of states of this system; it could be for example, the configuration of a robot (e.g., position and orientation of a mobile robot or joint configuration of a robotic manipulator) within a known environment (or within a set of possible environments), or it can be extended to include also the (higher order) derivatives of its configuration. See (LaValle, 2012, Section 3.1) for possible state spaces of a mobile robot. Next, let *U* be the set of control inputs (also referred to as actions). When applied at a state *x* ∈ *X*, a control *u* ∈ *U* causes *x* to change according to a state transition function *f* : *X* × *U* → *X*. Indeed, an action *u* ∈ *U* refers to the control input to the system and corresponds to the stimuli created by a control command generated by a decision maker. Mathematically, *the external system* can now be expressed as the triple (*X*, *U*, *f*). The sets *X* and *U* can be finite or infinite discrete spaces, or they could be equipped with extra structure: they could be metric manifolds, vector spaces, compact, or non-compact topological spaces. In such cases, the function *f* may or may not be assumed to respect such structure: sometimes it is appropriate to assume continuity or measurability.

The internal system (robot brain) corresponds to the perspective of a decision maker. The states of this system correspond to the retained information gathered through the outcomes of actions in terms of sensor observations. To this end, the basis of our mathematical formulation of the internal system is the notion of an *information space (I-space)* presented in (LaValle, 2006: Chapter 11). Let I be the set of these internal information states. We will use the term *information state (I-state)* to refer to elements of I and denote them by *ι*. Similar to the external system, the internal system evolves with each *y* ∈ *Y* according to the *information transition function* ϕ:I×Y→I . The *internal system* can now mathematically be described by the triple ( I,Y,ϕ ).

The external (*X*, *U*, *f*) and internal ( I,Y,ϕ ) can be *coupled* to each other to create a *coupled dynamical system*. This is achieved by introducing two coupling functions that match outputs of one system to the inputs of the other and vice versa. For us, these are the *sensor mapping h*: *X* → *Y* and the *policy* π:I→U , see Figure 1. The function *h* labels the external states with sensory data, and *π* labels the internal information states with the actions. Note that *π* can be seen as an information-feedback policy sending a control to the external. In the following parts of this paper, we will refer to *π* simply as the policy. Therefore, it will be a map from the states of an I-space (Section 3.2 and 3.4 will present possible I-space descriptions) to the set of controls. This definition is more general than the use of policy in the robotics literature which typically refers to a state-feedback policy, that is, a map from the states of a deterministic or a probabilistic description of the external system.

Suppose the system evolves in discrete stages. Then, the coupled dynamical system can be written as

(1) $$\begin{array}{l} \iota^{\prime }=\phi \left(\iota ,y\right)\;\text{in}\;\text{which}\;y=h\left(x\right),\\ x^{\prime }=f\left(x,u\right)\;\text{in}\;\text{which}\;u=\pi \left(\iota^{\prime }\right). \end{array}$$

Here we use *x*′ to refer to the next state, not the derivative of *x*. Whereas the equations on the left side describe the evolution of this coupled system, the ones on the right show the respective outputs of each subsystem. The coupled system of internal and external described this way is an autonomous system, meaning that given an initial state (ι,x)∈I×X there exists a unique state trajectory.^1^ We denote the function (*ι*, *x*)↦(*ι*′, *x*′) by ϕ*_π,*h*_ *f* which highlights that ϕ*_π,*h*_ *f* is a coupling of *ϕ* and *f* via the pair of coupling functions (*π*, *h*). Then, the coupled system is the pair

$$(I\times X,\phi {*}_{\pi ,h}\;f).$$

For the external system, starting from an initial state *x*_1_, each stage *k* corresponds to applying an action *u*_*k*_ which then yields the next stage *k* + 1 and the next state *x*_*k*+1_ = *f* (*x*_*k*_, *u*_*k*_). As the system evolves through stages, the tuples ${\tilde{x}}_{k}=\left(x_{1},x_{2},\ldots ,x_{k}\right)$ , ${\tilde{u}}_{k-1}=\left(u_{1},u_{2},\ldots ,u_{k-1}\right)$ and ${\tilde{y}}_{k}=\left(y_{1},y_{2},\ldots ,y_{k}\right)$ correspond respectively to the state, action, and observation histories up to stage *k*, with *y*_*i*_ = *h*(*x*_*i*_) for *i* ∈ {1, *…*, *k*}. Note that applying the action *u*_*k*_ at stage *k* would result in a transition to state *x*_*k*+1_ and the corresponding sensor reading *y*_*k*+1_ = *h*(*x*_*k*+1_). The same applies for the internal system. We can describe its evolution starting from an initial I-state *ι*_0_, and following the state transition equation *ι*_*k*_ = *ϕ*(*ι*_*k*−1_, *y*_*k*_). At stage *k*, *π*(*ι*_*k*_) produces the action *u*_*k*_. Note that the stage index of the I-state starts from 0. In some cases, *ι*_0_ can encode prior information regarding the external system and in others, it does not. We will consider this distinction more formally in Section 3.2. The next information state *ι*_1_ is obtained using *ι*_0_ and *y*_1_. We assume that no control command (action) is outputted at stage 0, meaning that the control history starts with *u*_1_. By convention, ${\tilde{u}}_{0}=\left(\right)$ is an empty sequence.

### 2.2. Disturbances

**Figure 2.**
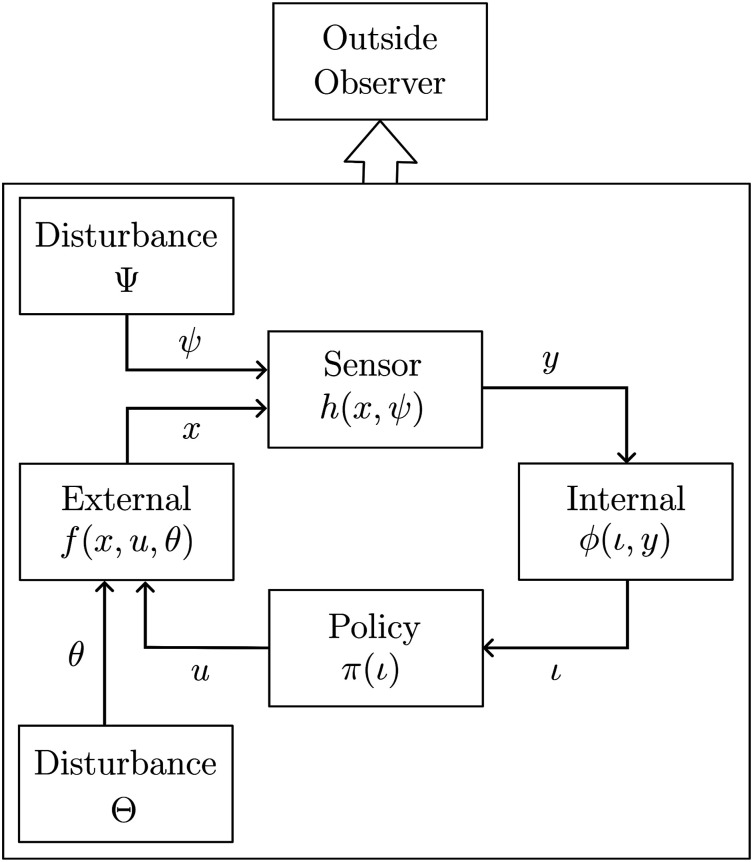
Disturbances may affect the external system and the sensor. Note that conditioned on the realization of these, the internal system and policy remain deterministic. An outside observer (planner/designer) may perceive the coupled system as a whole.

The coupled internal-external systems formulation can be extended to include disturbances affecting the external system and the sensor. In particular, we can define two disturbance generating systems, with outputs *θ* ∈ Θ and *ψ* ∈ Ψ, that are influencing the external system and the sensor, respectively (see Figure 2). Mathematically, the external system with disturbances is (*X*, *U* × Θ, *f*), where *f* : *X* × (*U* × Θ) → *X* is a state transition function for the external system under disturbances. Thus, the disturbances merely add a new dimension to the control parameters of the system. In the internal–external coupling, we also assume disturbances in the sensory mapping which takes the form h:X×Ψ→I . Then, the definition of the coupled internal–external system given in (1) is modified as follows:

(2) $$\begin{array}{l} \iota^{\prime }=\phi \left(\iota ,y\right)\;\text{in}\;\text{which}\;y=h\left(x,\psi \right),\\ x^{\prime }=f\left(x,u,\theta \right)\;\text{in}\;\text{which}\;u=\pi \left(\iota^{\prime }\right). \end{array}$$

Here, the other two functions *ϕ* (the information transition function) and *π* (policy) are as in Section 2.1.

Note that neither *θ*, which affects the state transition function of the external system, nor *ψ*, which affects the sensor mapping, is directly available to the internal system (I,Y,ϕ) , which is just as in Section 2.1. However, some information regarding the disturbances can be specified for an internal system that makes use of a model of the external. These could be encoded into the set I and the transition function *ϕ*. Then, the internal system has a nontrivial correlation with the disturbance, even though it is never directly perceived. We will consider the distinction between model-based and model-free systems in Section 3.5. Finally, the coupled system is mathematically a triple ( I×X,Θ×Ψ,g) where *g* is a function that, given a state (ι,x)∈I×X and a disturbance parameter (*θ*, *ψ*) ∈ Θ ×Ψ, outputs the next state in I×X , formally,

g:(I×X)×(Θ×Ψ)→I×X.

There are two possibilities for how the information regarding the disturbances can be specified: nondeterministic and probabilistic. In the nondeterministic case, the set Ψ and possibly a subset Ψ(*x*) ⊆Ψ is specified for all *x* ∈ *X*, in which Ψ(*x*) represents the set of all *ψ* that can be realized for each *x*. In the probabilistic case, assuming that the disturbances are generated by a system that is Markovian, such that they do not depend on the previous stages, a probability distribution over Ψ can be specified for each *x*. This will be denoted as *P*(*ψ*∣*x*). The disturbances affecting the external system can be specified similarly to those that affect the sensor. In the nondeterministic case, the set Θ(*x*, *u*) ∈ Θ is known for each (*x*, *u*) ∈ *X* × *U*. In the probabilistic case, a probability distribution over Θ, that is, *P*(*θ*∣*x*, *u*), can be specified for each (*x*, *u*).

### 2.3. Generalizing to transition systems

A *transition system* is a triple (*S*, Λ, *T*), in which, *S* and Λ are some sets (possibly equipped with some structure, for example, topology), and *T* ⊆ *S* × Λ × *S* is a ternary relation. Here *S* is the set of states, Λ is the set of labels for transitions between elements of *S*, and a triple (*s*, *λ*, *s*′) belongs to *T* if there is a transition from *s* to *s*′ labeled by *λ*. A special case is when for each (*s*, *λ*) ∈ *S* × Λ there is a unique *s*′ ∈ *S* with (*s*, *λ*, *s*′) ∈ *T*. Then, *T* defines a function *τ*: *S* × Λ → *S*. These are called *deterministic transition systems*, and sometimes also *(open) dynamical systems*. An extensive analysis of those and their coupling is explored by (Spivak, 2015).

All models in Sections 2.1 and 2.2 are deterministic transition systems: the external, the internal, the disturbed versions, and their couplings are all deterministic transition systems.^2^ Note that if Λ is a singleton, the system (*S*, Λ, *τ*) is equivalent to a discrete time autonomous dynamical system. If (*S*, Λ, *τ*) is a deterministic transition system, *S* and Λ are finite, *s*_0_ ∈ *S*, and *F* ⊆ *S*, then (*S*, Λ, *τ*, *s*_0_, *F*) is a finite automaton as defined in (Sipser, 2012, Definition 1.5). If not stated otherwise, we do not assume our systems to be finite. In Section 5.3, we explore connections between our theory with the theory of finite systems.

The following notion of state-relabeled transition systems was introduced in (Weinstein et al., 2022) to model the internal and external systems.

Definition 1. State-relabeled transition system A state-relabeled transition system is the quintuple (*S*, Λ, *T*, *σ*, *L*) in which *σ*: *S* → *L* is a labeling function and (*S*, Λ, *T*) is a transition system. The function *σ* is the *labeling function* and *L* is the set of labels.

A state-relabeled transition system is closely related to the *Moore machine* which is a state-relabeled transition system with a fixed initial state, a finite set of states, and finite sets of input and output alphabets (finite Λ and *L*) (Moore, 1956).

In our framework, a labeling function *σ* serves two purposes; it enables a potential coupling by matching output of one system to the input of another, and acts as a categorization of the states of the system being labeled. Preimages of a labeling function *σ* induce a partition of the state space *S* into sets whose elements are indistinguishable through sensing. Let *S*/*σ* be the set of equivalence classes [*s*]_*σ*_ induced by *σ* such that *S*/*σ* = {[*s*]_*σ*_∣*s* ∈ *S*} and [*s*]_*σ*_ = {*s*′ ∈ *S*∣*σ*(*s*′) = *σ*(*s*)}. Then, using these equivalence classes, we can define a new transition system called the *quotient* of (*S*, Λ, *T*) by *σ*.

Definition 2. Quotient system The quotient of (*S*, Λ, *T*) by *σ* is the transition system (*S*/*σ*, Λ, *T*/*σ*), in which

$$T/\sigma :=\left\{\left({\left[s\right]}_{\sigma },\lambda ,{\left[s^{\prime }\right]}_{\sigma }\right)|\left(s,\lambda ,s^{\prime }\right)\in T\right\}.$$

Note that (*S*/*σ*, Λ, *T*/*σ*) is a reduced version of (*S*, Λ, *T*), in the sense that the map *s*→[*s*]_*σ*_ is onto, but not necessarily one-to-one.^3^ We might be interested in finding a labeling function *σ* such that the corresponding quotient transition system is as simple as possible while ensuring that it is still useful. In the following sections, we will provide motivations for a reduction and discuss in more detail the requirements on *σ* for the quotient system to be useful.

The external and internal systems can be written as state-relabeled deterministic transition systems (*X*, *U*, *f*, *h*, *Y*) and (I,Y,ϕ,π,U) , respectively, in which *h* and *π* are considered as labeling functions. Interpreting the labels as the output of a transition system, a coupled internal–external system can be described in terms of the state-relabeled transition systems formulation too, so that the output of one transition system is an input for another. Described this way, a coupling of two transition systems results in unique paths in either transition system, initialized at a particular state.

## 3. Sufficient information transition systems

### 3.1. Information transition systems

In the general setting, an I-state corresponds to the available (stored) information at a certain stage with respect to the action and observation histories. An I-space is a collection of all possible I-states. We will use the acronym ITS to refer to an *information transition system*, that is, a transition system whose state space is an I-space.

We have already used the notion of an I-space when modeling the internal system representing the robot brain, which we view as an ITS. Here, we extend the notion of an ITS to include different perspectives from which the external and the coupled systems can be viewed. In particular, we identify three perspectives;

 • a planner,
 • a plan executor,
 • and an (independent) observer.

With a slight abuse of previously introduced notation and terminology, we use the term *internal* to refer to any system that is not the external system and we use I to denote a generic I-space. We use the term *deterministic information transition system* (DITS) to refer to an ITS for which the transitions are governed by an information transition function so that they are deterministic. We denote these types of systems by (I,Λ,ϕ) , in which Λ is the edge labeling and ϕ:I×Λ→I is an information transition function. Otherwise, an ITS will be called a *nondeterministic information transition system* (NITS) and denoted by (I,Λ,Φ) , in which Φ⊆I×Λ×I is the transition relation.

Suppose (I,U×Y,Φ) is a NITS and π:I→U a policy, and define

(3) $$\Phi_{\pi }:=\left\{\left(\iota ,\left(u,y\right),\iota^{\prime }\right)\in \Phi |u=\pi \left(\iota \right)\right\}\subseteq \Phi .$$

The transition system $\left(I,U\times Y,\Phi_{\pi }\right)$ is called the *restriction* of (I,U×Y,Φ) by the policy *π*.^4^ If (I,U×Y,ϕ) is a DITS, the *strong restriction* by π:I→U is given by $\left(I,Y,\phi_{\pi }\right)$ , in which $\phi_{\pi }:I\times Y\to I$ and *ϕ*_*π*_(*ι*, *y*) = *ϕ*(*ι*, *π*(*ι*), *y*). The strong restriction is obtained by first taking the restriction of *ϕ* treated as a subset of I×(U×Y)×I and then taking the projection of the resulting set onto I×Y×I .

Before any policy is fixed, a DITS of the form (I,U×Y,ϕ) corresponds to the *planner* perspective. Once the policy is fixed, the strong restriction $\left(I,Y,\phi_{\pi }\right)$ , which is just as the internal system was defined in Section 2, corresponds to the *plan executor*.

Example 1. A binary toy model Consider the DITS (I,U×Y,ϕ) which corresponds to a planner perspective. Suppose U=Y=I={0,1} and let ϕ:I×(U×Y)→I be defined by *ϕ*(*ι*, (*u*, *y*)) = |*y* − *u*|. Suppose a policy π:I→U is fixed such that *π*(*ι*) = *ι*. Then, $\left(I,Y,\phi_{\pi }\right)$ , in which *ϕ*_*π*_(*ι*, *y*) = |*y* − *π*(*ι*)|, is the strong restriction of (I,U×Y,ϕ) by *π*. Furthermore, it corresponds to the plan executor.

In this paper, an observation will refer to a sensor reading *y*. However, when we discuss an (independent) observer described over the coupled system, the input to this observer system can be a function of any variable of the coupled system, for instance action, information state or the state of the external. If the coupled system is disturbed, the disturbances can be observed by the observer too.

### 3.2. History information spaces

The most fundamental I-space is the *history I-space*, which we denote by $I_{hist}$ . A *history I-state* at stage *k* corresponds to all the information that is gathered through sensing (and potentially also through actions) up to stage *k*, assuming perfect memory. In this sense, $I_{hist}$ is the canonical I-space, and all the other I-spaces are derived from it. We denote the history I-states by the letter *η* to distinguish them from the states of other information spaces, which we typically denote by *ι* (recall the notation introduced in Section 2.1).

Let *U* and *Y* be the sets of possible actions and observations respectively. The elements of $I_{hist}$ are finite sequences of alternating actions and observations which build upon some initial state $\eta_{0}\in I_{hist}$ . Denote the set of possible initial states of $I_{hist}$ by $I_{0}$ . Then, the elements of $I_{hist}$ are of the form

(4) $$\left(\eta_{0},{\tilde{u}}_{k-1},{\tilde{y}}_{k}\right):=\left(\eta_{0},y_{1},u_{1},y_{2},u_{2}\ldots ,u_{k-1},y_{k}\right)$$

for k∈N , in which $\eta_{0}\in I_{0}$ , *u*_*i*_ ∈ *U,* and *y*_*i*_ ∈ *Y* for all *i* ≤ *k*. Additionally, denote

(5) $$\eta_{k}:=\left(\eta_{0},{\tilde{u}}_{k-1},{\tilde{y}}_{k}\right).$$

The notations (4) and (5) follow (LaValle, 2006: Chapter 11). The lower index *k* refers to the *stage* of the state, or the length of the action-observation sequence. The convention here, as already mentioned in the end of Section 2.1, is that ${\tilde{u}}_{0}$ is assumed to be the null-tuple. Thus, $\eta_{k}=\left(\eta_{0},{\tilde{u}}_{k-1},{\tilde{y}}_{k}\right)$ is the I-state at stage *k*, which is achieved by iteratively concatenating the action-observation pairs (*u*_*i*−1_, *y*_*i*_) at the end of the sequence for *i* ∈ {1, *…*, *k*} after the initial state *η*_0_.

The description of initial conditions in the set $I_{0}$ varies with the available prior information. We discuss these descriptions below. The history information space at stage *k* is the subset of $I_{hist}$ which consists of elements of the form given by (4) for fixed *k*, and can be expressed as the product

(6) $$I_{k}:=I_{0}\times {\tilde{U}}_{k-1}\times {\tilde{Y}}_{k},$$

in which ${\tilde{U}}_{k-1}=U^{k-1}$ and ${\tilde{Y}}_{k}=Y^{k}$ . In general, the number of stages that the system will go through is not fixed. Therefore, we assume the history I-space to contain all finite action-observation sequences, that is, $I_{hist}=\underset{k\in N}{\cup }I_{k}$ . The DITS corresponding to $I_{hist}$ is $\left(I_{hist},U\times Y,\phi_{hist}\right)$ , in which

$$\phi_{hist}\left(\eta ,u,y\right)=\eta^{⌢}\left(u,y\right),$$

${and}^{⌢}$ is the concatenation of two sequences. Note that the concatenation operation makes $\left(I_{hist}{,}^{⌢}\right)$ into a free monoid. The derived information transitions systems which will be introduced in Section 3.4 can be seen as quotients of this monoid by equivalence relations; sometimes these quotients can also be monoids, or even groups.

### 3.3. Sufficient state-relabeling

In (Weinstein et al., 2022), we have introduced a notion of *sufficiency* that generalizes the definition introduced in (LaValle, 2006: Chapter 11) and is presented here for completeness.

Definition 3. Sufficient labeling function Let (*S*, Λ, *T*) be a transition system. A labeling function *σ*: *S* → *L* defined over the states of a transition system is *sufficient* if and only if for all *s*, *t*, *s*′, *t*′ ∈ *S* and all *λ* ∈ Λ, the following implication holds:

$$\begin{array}{l} \sigma \left(s\right)=\sigma \left(t\right)\wedge \left(s,\lambda ,s^{\prime }\right)\in T\wedge \left(t,\lambda ,t^{\prime }\right)\in T\Rightarrow \\ \sigma \left(s^{\prime }\right)=\sigma \left(t^{\prime }\right). \end{array}$$

If *σ* is defined over the states of a deterministic transition system (*S*, Λ, *τ*), then *σ* is sufficient if and only if for all *s*, *t* ∈ *S* and all *λ* ∈ Λ, *σ*(*s*) = *σ*(*t*) implies that *σ*(*τ*(*s*, *λ*)) = *σ*(*τ*(*t*, *λ*)).

Consider the stage-based evolution of the state-relabeled deterministic transition system corresponding to the external system (*X*, *U*, *f*, *h*, *Y*) with respect to the action (control input) sequence ${\tilde{u}}_{k-1}=\left(u_{1},\ldots ,u_{k-1}\right)$ . This corresponds to the state and observation histories till stage *k*, that are ${\tilde{x}}_{k}=\left(x_{1},\ldots ,x_{k}\right)$ and ${\tilde{y}}_{k}=\left(y_{1},\ldots ,y_{k}\right)$ . Recall that applying *u*_*k*_ at stage *k* would result in a transition to *x*_*k*+1_ and the corresponding observation *y*_*k*+1_ = *h*(*x*_*k*+1_). Hence, in this context, sufficiency of *h* implies that given the label *y*_*k*_ = *h*(*x*_*k*_) and the action *u*_*k*_, it is possible to determine the label *y*_*k*+1_ = *h*(*x*_*k*+1_). One interpretation of sufficiency of *h* is that the respective quotient system sufficiently represents the underlying system up to the equivalence classes induced by *h*. This notion is similar to a minimal realization of a system, that is, the minimal state space description that models the given input-output measurements (see for example (Kotta et al., 2018)). Another interpretation is in a predictive sense. Suppose the quotient system is known. Then, the label *y*_*k*+1_ = *h*(*x*_*k*+1_) can be determined before the system gets to *x*_*k*+1_, using the current label *y*_*k*_ and the action to be applied *u*_*k*_. Furthermore, under a fixed policy, the complete observation trajectory can be determined from the initial observation by induction.

Now, consider an internal system with a labeling function $\kappa :I\to I^{\prime }$ , that is, $\left(I,U\times Y,\phi ,\kappa ,I^{\prime }\right)$ , and its evolution with respect to the histories $\tilde{y}=\left(y_{1},\ldots ,y_{k}\right)$ and $\tilde{u}=\left(u_{1},\ldots ,u_{k-1}\right)$ . At stage *k*, the state of the DITS is *ι*_*k*_ and with (*u*_*k*_, *y*_*k*+1_) the system transitions to *ι*_*k*+1_ = *ϕ*(*ι*_*k*_, *u*_*k*_, *y*_*k*+1_). Sufficiency of *κ* implies that given *κ*(*ι*_*k*_), *u*_*k*_, and *y*_*k*+1_, we can determine *κ*(*ι*_*k*+1_). This is equivalent to the definition introduced in (LaValle, 2006: Chapter 11) and makes it a special case of Definition 3.

### 3.4. Derived information transition systems

Even though it seems natural to rely on a history ITS, the dimension of a history I-state increases linearly, and the size of the history I-space increases exponentially, as a function of the stage index, making it impractical in most cases. Thus, we are interested in defining a reduced ITS that is more manageable, due to, for example, lowered requirements for memory or computing power. Furthermore, this would largely simplify the description of a policy for a planner or a plan executor.

Recall the quotient of a transition system by a labeling function (see Definition 2). We rewrite $\left(I_{hist},U\times Y,\phi_{hist}\right)$ as $\left(I_{hist},U\times Y,\Phi_{hist}\right)$ , in which

(7) $$\begin{array}{l} \Phi_{hist}=\{\left(\eta ,\left(u,y\right),\eta \prime \right)\in \\ I_{hist}\times \left(U\times Y\right)\times I_{hist}|\eta \prime =\phi_{hist}\left(\eta ,u,y\right)\}. \end{array}$$

We can introduce an *information mapping* (I-map) $\kappa :I_{hist}\to I_{der}$ that categorizes the states of $I_{hist}$ into equivalence classes through its preimages. In this case, *κ* serves as a labeling function. A reduction is obtained in terms of the quotient of $\left(I_{hist},U\times Y,\Phi \right)$ by *κ*, that is, $\left(I_{hist}/\kappa ,U\times Y,\Phi /\kappa \right)$ as histories are grouped into equivalence classes.

It is crucial that the derived ITS is a DITS so that the transition from the current label to the next can be determined using only the derived ITS, without making reference to the history ITS. The reason for this requirement is straightforward for an observer as the I-states correspond to what is inferred about the external system, given observation history (potentially accompanied by the action history). The same applies for the planner and the plan executor to be able to describe and execute a policy. Considering the quotient system derived by *κ* from the DITS (by definition) $\left(I_{hist},U\times Y,\phi \right)$ , we cannot always guarantee that the resulting ITS is deterministic. This depends on the I-map used for state-relabeling, as illustrated in the following proposition.

Proposition 1 **Quotient of a history ITS may be a NITS**. *For all non-empty U and Y, and for the corresponding* $I_{hist}$ *, there exists a labeling function κ such that the quotient* $\left(I_{hist}/\kappa ,U\times Y,\Phi /\kappa \right)$ *of* $\left(I_{hist},U\times Y,\phi \right)$ *by κ, in which* Φ *is defined as in* (7)*, is not a DITS.*

**Proof**. Let $\kappa :I_{hist}\to \left\{l_{1},l_{2}\right\}$ and define $\kappa^{-1}\left(l_{1}\right)=\left\{\eta_{k}=\left({\tilde{u}}_{k-1},{\tilde{y}}_{k}\right)\in I_{hist}|{\tilde{u}}_{k-1}={\left(u_{i}\right)}_{i=1}^{k-1},u_{i}=u\;\text{for}\;1\leq i\leq k-1,\text{and}\;k>3\right\}$ , and $\kappa^{-1}\left(l_{2}\right)=I_{hist}\setminus \kappa^{-1}\left(l_{1}\right)$ . Then *κ*^−1^(*l*_1_) is the set of histories of length *k* > 3 which correspond to applying the same action *u* for *k* − 1 times, and *κ*^−1^(*l*_2_) is its complement. Then, there exist sequences $\eta_{k-2}=\left({\tilde{u}}_{k-3},{\tilde{y}}_{k-2}\right)$ and $\eta_{k-1}=\left({\tilde{u}}_{k-2},{\tilde{y}}_{k-1}\right)$ such that *η*_*k*−2_ = *η*_*k*−1_^⌢^(*u*, *y*) and *η*_*k*_ = *η*_*k*−1_^⌢^(*u*, *y*) for which *κ*(*η*_*k*−2_) = *κ*(*η*_*k*−1_) = *l*_2_ and *κ*(*η*_*k*_) = *l*_1_. Thus,

$$\left\{\left({\left[\eta_{k-2}\right]}_{\kappa },\left(u,y\right),{\left[\eta_{k-1}\right]}_{\kappa }\right),\left({\left[\eta_{k-1}\right]}_{\kappa },\left(u,y\right),{\left[\eta_{k}\right]}_{\kappa }\right)\right\}\in \Phi /\kappa .$$

Since ${\left[\eta_{k-2}\right]}_{\kappa }={\left[\eta_{k-1}\right]}_{\kappa }$ and ${\left[\eta_{k-1}\right]}_{\kappa }\neq {\left[\eta_{k}\right]}_{\kappa }$ , the transition corresponding to $\left({\left[\eta_{k-1}\right]}_{\kappa },\left(u,y\right)\right)$ is not unique; thus, $\left(I_{hist}/\kappa ,U\times Y,\Phi /\kappa \right)$ is not deterministic.

■

Note that Proposition 1 holds also in the case of a generic ITS (I,U×Y,ϕ) , with non-history I-states, if there exist $s,s^{\prime },q,q^{\prime }\in I$ such that {(*s*, (*u*, *y*), *s*′), (*q*, (*u*, *y*), *q*′)} ∈ Φ, in which Φ is defined using *ϕ* as in (7). Then, any I-map *κ* such that *κ*(*s*) = *κ*(*q*) and *κ*(*s*′) ≠ *κ*(*q*′) results in a quotient system that is not a DITS.

Remark 1 *Whether the quotient system derived from* $\left(I_{hist},U\times Y,\phi \right)$ *is a DITS depends on the sufficiency of κ. In (*Weinstein et al., 2022*, Proposition 4.5) it is shown that the quotient of a transition system* (*S*, Λ, *T*) *by a labeling function σ is a deterministic transition system if and only if* (*S*, Λ, *T*) *is full* ^5^ *and σ is sufficient.*

As *ϕ*_*hist*_ is a function with domain $I_{hist}\times \left(U\times Y\right)$ , it is full, so the following is implied by (Weinstein et al., 2022) as a special case:

Proposition 2 **A Quotient system is a DITS when the labeling is sufficient.*** Let* $\left(I_{hist}/\kappa ,U\times Y,\Phi_{hist}/\kappa \right)$ *be the quotient of* $\left(I_{hist},U\times Y,\phi_{hist}\right)$ *by κ, in which* Φ_*hist*_ *is defined as in* (7)*. Then*, $\left(I_{hist}/\kappa ,U\times Y,\Phi_{\mathrm{hist}}/\kappa \right)$ *is a DITS if and only if κ is sufficient*.

Remark 2 *For an I-map* $\kappa :I_{hist}\to I_{der}$ *, the quotient* $\left(I_{hist}/\kappa ,U\times Y,\Phi_{hist}/\kappa \right)$ *is isomorphic to* $\left(I_{der},U\times Y,\phi_{der}\right)$ *, in which*

$$\Phi_{der}=\left\{\left(\kappa \left(\eta \right),\left(u,y\right),\kappa \left(\eta^{\prime }\right)\right)\;|\left(\eta ,\left(u,y\right),\eta^{\prime }\right)\in \Phi_{hist}\right\}$$

(Weinstein et al., 2022, *Proposition 2.37*). *Thus, we can use the labels introduced by an κ as the new* (*derived*) *I-space and the corresponding quotient system as the derived ITS.*

Suppose an I-map *κ* is sufficient. Then, the derived ITS is a DITS, meaning that given an I-state *ι*_*k*−1_ in the derived space $I_{der}$ , and (*u*_*k*−1_, *y*_*k*_), $\iota_{k}\in I_{der}$ can be uniquely determined. Consequently, we can write the derived ITS as $\left(I_{der},U\times Y,\phi_{der}\right)$ in which

$$\phi_{der}:I_{der}\times \left(U\times Y\right)\to I_{der}$$

is the new information transition function. Therefore, we no longer need to rely on the full histories and the history ITS and can rely solely on the derived ITS. This is shown in the first two rows of the following diagram:

(8) $$\begin{array}{cccccccc} \underset{↓\kappa }{I_{hist}} & \overset{u_{1},\;y_{2}}{\to } & \underset{↓\kappa }{I_{hist}} & \overset{u_{2},\;y_{3}}{\to } & \underset{↓\kappa }{I_{hist}} & \overset{u_{3},\;y_{4}}{\to } & \underset{↓\kappa }{I_{hist}} & \ldots \\ \underset{↓\kappa \prime }{I_{der}} & \overset{u_{1},\;y_{2}}{\to } & \underset{↓\kappa \prime }{I_{der}} & \overset{u_{2},\;y_{3}}{\to } & \underset{↓\kappa \prime }{I_{der}} & \overset{u_{3},\;y_{4}}{\to } & \underset{↓\kappa \prime }{I_{der}} & \ldots \\ \underset{↓\kappa \prime \prime }{I_{\min}} & \overset{u_{1},\;y_{2}}{\to } & \underset{↓\kappa \prime \prime }{I_{\min}} & \overset{u_{2},\;y_{3}}{\to } & \underset{↓\kappa \prime \prime }{I_{\min}} & \overset{u_{3},\;y_{4}}{\to } & \underset{↓\kappa \prime \prime }{I_{\min}} & \ldots \\ I_{task}\; & & I_{task}\; & & I_{task}\; & & I_{task} & \ldots \end{array}$$

Note that we can similarly define an I-map that maps any derived I-space to another. An example is given in (8) as the mappings $\kappa \prime :I_{der}\to I_{\min}$ and $\kappa \prime \prime :I_{\min}\to I_{task}$ . In this example, *κ*′ is sufficient, visible also from the commutativity of the respected square in the diagram. This implies that the quotient system derived by *κ*′ is deterministic. On the other hand, *κ*″ is not sufficient, meaning that the derived ITS is not deterministic: given an element of $I_{task}$ one cannot uniquely determine the next I-state using the derived ITS only. This is shown in (8) with the missing arrows at the respective row of $I_{task}$ . Hence, for *κ*″ the diagram does not commute. Note that an I-map whose domain is $I_{hist}$ can also be defined as composition of the mappings along the column of the diagram. For example, $\kappa_{\min}:I_{hist}\to I_{\min}$ is the composition of *κ* and *κ*′, that is, *κ*_min_ = *κ*′∘*κ* (same for $\kappa_{task}:I_{hist}\to I_{task}$ ).

### 3.5. Model-based and model-free

In machine learning, control, and robotics literature, methods are often categorized into *model-based* and *model-free* (or data-driven) ones. Informally, using our setup, a model-based scenario is one where the derived I-state is allowed to depend on knowledge about the external system, the sensor mapping, the initial state, and the disturbances acting on the external system or on the sensors (if there are any). The model-free scenario in contrast cannot depend on those, but can depend on data, which in our case is the history I-states, that is, the sequences of actions and observations.

In Section 2.1 we have defined internal-external coupled systems. Their coupling (1) produces an autonomous system $\left(I\times X,\phi \;{*}_{\pi ,h}\;f\right)$ . However, we can choose not to consider either one of the coupling functions *π* and *h*, and be left with a system that still has a control parameter. For example, let $\left(I\times X,U,\phi \;{*}_{h}\;f\right)$ be a system where the evolution of states can be written as

(9) $$\begin{array}{l} \iota \prime =\phi \left(\iota ,y\right)\;\text{in}\;\text{which}\;y=h\left(x\right),\\ x\prime =f\left(x,u\right). \end{array}$$

Here, *u* ∈ *U* is a control parameter on which the next state always depends. This system represents the coupled system before a policy *π* has been defined over the states of the internal.

Note that the internal system only has access to the current information state ι∈I , not to the external state *x* ∈ *X*. One can notationally express this perspective by evolving the internal system by an externally parametrized information transition function *ϕ*_*f*,*h*_( ⋅ ; *x*), which maps the current I-state and action pair (ι,u)∈I×U to the next I-state ι′∈I . The maps *h* (which couples the external to the internal system) and *f* are subsumed into the global map *ϕ*_*f*,*h*_ which is additionally parametrized by the current state *x* ∈ *X* of the external system. Thus, in accordance with (9), we define *ϕ*_*f*,*h*_ for each (ι,u)∈I×U and *x* ∈ *X* by

(10) $$\phi_{f,h}\left(\iota ,u;x\right):=\phi \left(\iota ,h\left(f\left(x,u\right)\right)\right).$$

If the I-space in (9) is the history I-space, we can write (9) as $\left(I_{hist}\times X,U,\phi \;{*}_{h}f\right)$ , and its internal system perspective (10) becomes $\left(I_{hist},U,\phi_{f,h}\right)$ . We propose that a method of obtaining a derived I-space corresponding to an I-map *κ* is *model-based*, if *κ* is obtained as a function of $\left(I_{hist}\times X,U,\phi \;{*}_{h}f\right)$ , while it is *model-free*, if it is obtained as a function of it from the perspective of the internal system, that is, as a function of $\left(I_{hist},U,\phi_{f,h}\right)$ .

The distinction between model-based and model-free is also seen in the initial states *η*_0_ of the history I-space. In model-based setups, typically *η*_0_ is a subset of *X*, or a probability distribution over *X* while in model-free setups *η*_0_ is an empty sequence. Examples 9 and 11 are examples of model-free and model-based I-spaces respectively.

Note that this formalization implies that model-free methods are a subset of model-based. This is because the internal perspective is itself a function of the entire coupled system, so anything that is a function of the internal perspective is by transitivity also a function of the entire coupled system. This matches the intuition that model-based are ones where more information is available. We leave the exploration of more aspects of this distinction and its formalization for future work.

We now present two examples that illustrate model-based and model-free derived ITSs.

Example 2. Bayesian filter Suppose the initial history information state encodes a probability distribution over *X* such that *η*_0_ = *P*(*x*_1_). We refer to the coupled system including the disturbances described in (2). A Markovian, probabilistic model of the disturbances is given in the form of conditional distributions *P*(*ψ*∣*x*) over Ψ, and *P*(*θ*∣*x*, *u*) over Θ. In the former, conditioning takes place relative to external states *x* ∈ *X*, and in the latter relative to state-action pairs (*x*, *u*) ∈ *X* × *U*. Using the definitions of *f* and *h* given in (2), *P*(*y*_*k*_∣*x*_*k*_) and *P*(*x*_*k*+1_∣*x*_*k*_, *u*_*k*_) can be derived from *P*(*ψ*_*k*_∣*x*_*k*_) and *P*(*x*_*k*+1_∣*x*_*k*_, *u*_*k*_) for all stages *k*.

Let $I_{prob}$ be the set of all probability distributions defined over *X* and let $I_{hist}$ be a history information space with $I_{0}=I_{prob}$ such that *η*_0_ is a probability distribution over *X*, that is, *P*(*x*_1_). An ITS can be derived by $\kappa_{prob}:I_{hist}\to I_{prob}$ such that *κ*_*prob*_(*η*_*k*_) = *ι*_*k*_ = *P*(*x*_*k*_∣*η*_*k*_). Note that we can write *η*_*k*_ as *η*_*k*_ = *η*_*k*−1_^⌢^(*u*_*k*−1_, *y*_*k*_). The I-state *ι*_*k*_ = *P*(*x*_*k*_∣*η*_*k*_) can be inductively computed from *ι*_*k*−1_ and (*u*_*k*−1_, *y*_*k*_) using marginalization and Bayes’ rule starting from *ι*_1_ = *P*(*x*_1_∣*y*_1_), in which *η*_1_ = *y*_1_. This corresponds to defining ϕ:I×(U×Y)→I such that *ι*_*k*_ = *ϕ*(*ι*_*k*−1_, (*u*_*k*−1_, *y*_*k*_)). Then, *κ*(*η*_*k*−1_^⌢^(*u*_*k*−1_, *y*_*k*_)) = *ϕ*∘*κ*(*η*_*k*−1_) = *P*(*x*_*k*_∣*η*_*k*_) which shows that *κ*_*prob*_ is sufficient. Hence, a Bayesian filter can be modeled as a derived DITS whose state space is $I_{prob}$ . Note that in this case, *κ*_*prob*_ is defined as a function of $\left(I_{hist}\times X,U,\phi \;{*}_{h}\;f\right)$ , making it model-based.

Note that the Kalman filter is a special case of a Bayesian filter when *f* and *h* are linear and the disturbances are Gaussian. These specifications imply that all the posterior distributions are Gaussian as well. Therefore, in this special case, the range of *κ*_*prob*_ is implicitly restricted to the set of all Gaussian distributions, denoted as $I_{Gauss}$ , such that $\kappa_{prob}:I_{hist}\to I_{Gauss}\subset I_{prob}$ . This restriction allows the I-state to simply encode only the mean and the covariance of a multivariate Gaussian distribution, that is, $\iota =\left(\hat{x},\Sigma \right)$ , in which $\hat{x}$ is the mean and Σ is the covariance matrix, without violating the sufficiency of *κ*_*prob*_. An extension of the Kalman filter to nonlinear systems is the Extended Kalman Filter (EKF). In the case of EKF, the functions *f* and *h* are not linear. This violates the posterior distribution being Gaussian even if the disturbances are. However, the states of the EKF are defined as elements of $I_{Gauss}$ and a state transition function $\phi :I_{Gauss}\times \left(U\times Y\right)\to I_{Gauss}$ is described that relies on linearizing *f* and *h* at each I-state. Note that even though the Kalman filter and the EKF share the same underlying I-space, the corresponding I-maps that derive these transition systems are different.

The following is an example of a model-free derived ITS.

Example 3. Moving average filter Let Y=R and $\kappa_{k}:I_{k}\to R$ , in which $I_{k}$ is the set of *k* stage histories. A moving average filter (observation only) with a window size *n* can be derived from $I_{k}$ as

$$\left({\tilde{u}}_{k-1},{\tilde{y}}_{k}\right)\mapsto \frac{1}{n}\overset{k}{\underset{i=k-n+1}{\sum }}y_{i}.$$

### 3.6. Lattice of information transition systems

We fix $I_{hist}$ , which corresponds to fixing the set of initial states $I_{0}$ . Then, each I-map *κ* defined over $I_{hist}$ induces a partition of $I_{hist}$ through its preimages, denoted as $I_{hist}/\kappa$ .

Definition 4. Refinement of an I-map An I-map *κ*′ is a *refinement* of *κ*, denoted as *κ*′⪰*κ*, if $\forall A\in I_{hist}/\kappa \prime$ there exists a $B\in I_{hist}/\kappa$ such that *A* ⊆ *B*.

Let $K\left(I_{hist}\right)$ denote the set of all partitions over $I_{hist}$ . Refinement induces a partial ordering since not all partitions of $I_{hist}$ are comparable. The partial ordering given by refinements form a lattice of partitions over $I_{hist}$ , denoted as ( $K\left(I_{hist}\right),≽)$ .

At the top of the lattice, there is the partition induced by an identity I-map (or equivalently, by a bijection), $\kappa_{id}:I_{hist}\to I_{hist}$ , since all of its elements are singletons (all equivalence classes contain exactly one element), making it the maximally distinguishable case. Conversely, we can define a constant mapping $\kappa_{const}:I_{hist}\to I_{const}$ for which $I_{hist}/\kappa_{const}$ is a singleton, that is, $I_{const}=\left\{\iota_{const}\right\}$ , which then will be at the bottom of the lattice. In turn, *κ*_*const*_ yields the minimally distinguishable case as all histories now belong to a single equivalence class. This idea is similar to the notion of the *sensor lattice* defined over the partitions of *X* (LaValle, 2012; Zhang and Shell, 2021). Indeed, if we take $I_{0}=X$ and consider $\kappa_{est}:I_{hist}\to X$ , the ordering of partitions of $I_{hist}$ such that $I_{hist}/\kappa_{est}$ is the least upper bound gives out the sensor lattice.

As motivated in previous sections, we are interested in finding a sufficient I-map such that the quotient ITS derived from the history ITS is still deterministic. Notice that the constant I-map *κ*_*const*_ is sufficient by definition since for all (*u*, *y*) ∈ *U* × *Y*, and all $\eta ,\eta \prime \in I_{hist}$ , we have that *κ*_*const*_(*η*) = *κ*_*const*_(*η*′) and $\kappa_{const}\left(\phi_{hist}\left(\eta ,\left(u,y\right)\right)\right)=\kappa_{const}(\phi_{hist}\left(\eta \prime ,\left(u,y\right)\right))$ . On the other hand, in certain cases, it is crucial to differentiate certain histories from others. This will become clear in the next section when we describe the notion of a task. Suppose *κ* is a labeling that partitions $I_{hist}$ into equivalence classes that are of importance and suppose that *κ* is not sufficient. Then, we want to find a refinement of *κ* that is sufficient. This will serve as a lower bound on the lattice of partitions over $I_{hist}$ since for any partition such that $I_{hist}/\kappa$ is a refinement of it, the classes of histories that are deemed crucial will not be distinguished. The following defines the refinement of *κ* that ensures sufficiency and a minimal number of equivalence classes.

Definition 5. Minimal sufficient refinement Let $\left(I_{hist},U\times Y,\phi_{hist}\right)$ be a history ITS and *κ* an I-map. A *minimal sufficient refinement* of *κ* is a sufficient I-map *κ*′ such that there does not exist a sufficient I-map *κ*″ that satisfies *κ*′ ≻ *κ*″⪰*κ*.

Remark 3 *It is shown in (*Weinstein et al., 2022*, Theorem 4.19) that the minimal sufficient refinement of κ defined over the states of a deterministic transition system* (*S*, Λ, *τ*) *is unique up to relabeling, namely if κ*_min_ *and* $\kappa \prime_{\min}$ *are minimal sufficient refinements, then* $\kappa_{\min}≻\kappa \prime_{\min}$ *and* $\kappa \prime_{\min}≻\kappa_{\min}$ .

## 4. Solving tasks minimally

### 4.1. Definition of a task

In this section, we formulate general planning and filtering tasks within the framework of information transition systems. We distinguish between two categories: 1) *active*, which entails planning and executing an information-feedback policy that forces a desirable outcome in the external system, and 2) *passive*, which refers to only observing the external system without being able to effect changes. We next describe active and passive tasks for the model-free and model-based I-space formulations, introduced in Section 3.5. In the model-free case, tasks are specified using a logical language over $I_{hist}$ . This results in a labeling, a derived I-space $I_{task}$ , and the associated I-map *κ*_*task*_. Various logics are allowable, such as propositional, modal, or a temporal logic. The resulting sentences of the language involve combinations of predicates that assign true or false values to subsets of $I_{hist}$ . Solving an active task requires that a sentence of interest becomes true during execution of the policy. This is called *satisfiability*. For example, the task may be to simply reach some goal set $G\subseteq I_{hist}$ , causing a predicate in-goal $\left(I_{hist}\right)$ to become satisfied (in other words, be true).

Solving a passive task only requires maintaining whether a sentence is satisfied, rather than forcing an outcome; this corresponds to filtering. Whether the task is active or passive, if satisfiability is concerned with a single, fixed sentence, then a *task-induced labeling* (or *task labeling* for short), that is, *κ*_*task*_, over $I_{hist}$ assigns two labels: Those I-states that result in true and those that result in false. A task labeling may also be assigned for a set of sentences. In this case, each sentence induces a partition of $I_{hist}$ , and the task labeling over $I_{hist}$ assigns a label to each set in the common refinement of these partitions. In the model-based case, tasks are instead specified using a language over *X*, and sentence satisfiability must be determined by an I-map that converts history I-states into expressions over *X*.

Some naturally occurring robot tasks can only be described in terms of infinite sequences of actions and observations. These are called *infinitary tasks*. For example, cycling through a finite sequence of subsets of *X* indefinitely while avoiding others (Fainekos et al., 2009) can only be described in terms of infinite histories. For this task, whether the sentence of interest is satisfied cannot be determined based on a finite history of any given length. However, the histories that fail, that is, those for which the sentence of interest becomes false, can be defined in terms of finite histories (namely those that result in a state that needed to be avoided). The interested reader can refer to (Kress-Gazit et al., 2009) for examples based on linear temporal logic (LTL).

Infinitary tasks are defined on the set of infinite histories $I_{hist}^{\infty }$ which consists of infinite sequences of the form $\bar{\eta }=\left(\eta_{0},y_{1},u_{1},y_{2},u_{2},\cdots \right)$ . These are the elements of the infinite Cartesian product

$$I_{0}\times \left(Y\times U\right)\times \left(Y\times U\right)\times \cdots =I_{0}\times \overset{\infty }{\underset{k=1}{\prod }}\left(Y\times U\right).$$

The preimages of an *infinitary task labeling* $\bar{\kappa }:I_{hist}^{\infty }\to I_{task}$ are subsets of $I_{hist}^{\infty }$ . Although the satisfiability of an infinitary task may depend on infinite sequences, these can nevertheless be characterized in terms of finite initial segments as follows. Any subset $H\subseteq I_{hist}^{\infty }$ can be written as

(11) $$H=I_{0}\times \overset{\infty }{\underset{k=1}{\prod }}\left(Y_{k}\times U_{k}\right),$$

in which $I_{0}\subseteq I_{0}$ , and *Y*_*k*_ ⊆ *Y*, *U*_*k*_ ⊆ *U* for all k∈N . For each m∈N , we denote by *H*(*m*) the collection of subsets of $I_{hist}^{\infty }$ for which *Y*_*k*_ = *Y* and *U*_*k*_ = *U* for all *k* > *m*, that is,

$$\begin{array}{l} H\left(m\right)=\{I_{0}\times \overset{m}{\underset{k=1}{\prod }}\left(Y_{k}\times U_{k}\right)\times \overset{\infty }{\underset{k=m+1}{\prod }}\left(Y\times U\right)|I_{0}\subseteq I_{0},Y_{k}\subseteq Y,U_{k}\subseteq U,k=1,\ldots ,m\}. \end{array}$$

In other words, such collections of histories are constrained only at a finite number of stages.

Now, let $\iota \in I_{task}$ and suppose an equivalence class induced by the preimage ${\bar{\kappa }}^{-1}\left(\iota \right)$ is a (potentially infinite) union

(12) $${\bar{\kappa }}^{-1}\left(\iota \right)=\underset{\alpha \in A}{\cup }H_{\alpha },$$

in which *A* is some index set and each *H*_*α*_ belongs to *H*(*m*) for some *m*. Then, whether a particular history $\bar{\eta }\in I_{hist}^{\infty }$ belongs to ${\bar{\kappa }}^{-1}\left(\iota \right)$ is determined by a finite number of stages in an initial segment of this history. In general, however, the length of these initial segments is not bounded from above.

To characterize infinitary tasks in terms of deciding their satisfiability, we rely on topology. Assume that some topology is defined for the sets $I_{0}$ , *Y*, and *U*. If these are finite sets, a natural choice is the discrete topology in which every singleton is an open set. For subsets of $R^{n}$ , a natural choice would be the relative topology induced by the usual Euclidean topology in $R^{n}$ . The base *H*° of the product topology in $I_{hist}^{\infty }$ consists of those sets *H* for which *H* ∈ *H*(*m*) for some m∈N , and the sets *I*_0_, *Y*_*k*_, *U*_*k*_ in (11) satisfy that *I*_0_ is open in $I_{0}$ , and *Y*_*k*_, *U*_*k*_ are open in *Y*, *U*, respectively, for all k∈N . All other open sets are obtained as arbitrary unions of sets *H* ∈ *H*°. In particular, when the sets *H*_*α*_ in (12) satisfy *H*_*α*_ ∈ *H*° for all *α* ∈ *A*, the corresponding preimage ${\bar{\kappa }}^{-1}\left(\iota \right)$ is an open set. If the sets $I_{0}$ , *Y*, *U* are compact, the space. $I_{hist}^{\infty }$ is compact in the product topology. This is the case for example when I , *Y*, and *U* are finite sets with the discrete topology.

In the simplest nontrivial case, a task labeling $\bar{\kappa }$ concerns a single sentence. Then, $I_{task}=\left\{0,1\right\}$ so that the preimages of $\bar{\kappa }$ partition $I_{hist}^{\infty }$ into the equivalence classes ${\bar{\kappa }}^{-1}\left(1\right)$ , that is, the set of histories for which the sentence is satisfied, and ${\bar{\kappa }}^{-1}\left(0\right)$ , the set of histories for which the sentence is false. We call ${\bar{\kappa }}^{-1}\left(1\right)$ and ${\bar{\kappa }}^{-1}\left(0\right)$ the *success set* and *fail set*, respectively. If the success set of a given task is open, we call this an *open task*. A *closed task* is one whose fail set is open, so that its success set is closed. It is possible for a task to be both open and closed, so that both the success and fail sets are both open and closed. We call such tasks *clopen*.

Due to the definition of the sets *H*(*m*), the membership of a given history in an open set is determined by some finite initial segment of that history. Therefore, based on a finite length segment of a given history, we can determine its membership in the success set of an open task, in the fail set of a closed task, and both the success and fail sets for a clopen task. In these cases, task satisfiability can be defined in terms of the elements of $I_{hist}$ . We can thus transcribe an infinitary task labeling $\bar{\kappa }:I_{hist}^{\infty }\to I_{task}$ in the form of $\kappa :I_{hist}\to I_{task}$ . This amounts to assigning to each finite history $\eta \in I_{hist}$ some task label $\iota \in I_{task}$ in such a way that this labeling expresses the success set of the corresponding infinitary task labeling $\bar{\kappa }$ . Recall that $I_{hist}=\cup_{k\in \mathbb{N}}I_{k}$ , in which $I_{k}$ are as in (6). Suppose ${\bar{\kappa }}^{-1}\left(\iota \right)$ is an open set for some $\iota \in I_{task}$ so that ${\bar{\kappa }}^{-1}\left(\iota \right)=\cup_{\alpha \in A}H_{\alpha }$ as in (12), with *H*_*α*_ open for all *α* ∈ *A*. We may assume that *H*_*α*_ ∈ *H*° for all *α* ∈ *A*. Then, for a finite history $\eta =\left(\eta_{0},y_{1},u_{1},\ldots ,u_{k-1},y_{k}\right)\in I_{k}$ we define *κ*(*η*) = *ι* if and only if there exists some *m* ≤ *k* and some index *α* ∈ *A* for which the corresponding set

$$H_{\alpha }=I_{0}\times \left(Y_{1}\times U_{1}\right)\times \cdots \times \left(Y_{m}\times U_{m}\right)\times \overset{\infty }{\underset{n=m+1}{\prod }}\left(Y\times U\right),$$

satisfies *y*_*n*_ ∈ *Y*_*n*_ and *u*_*n*_ ∈ *U*_*n*_ for all 1 ≤ *n* ≤ *m*.

Below are examples of typical model-based task descriptions with their corresponding definitions in terms of infinite histories that can be expressed using a task-labeling over $I_{hist}$ .

Example 4. Reach state ***x*** ∈ ***X*** from an initial state ***x*** _**0**_ ∈ ***X*
** The success set of this task consists of those histories that correspond to the external system arriving to *x* in some finite time. If $H_{m}\subseteq I_{hist}^{\infty }$ contains those histories in which *x* is visited for the first time at stage *m*, the success set of this task is ${\bar{\kappa }}^{-1}\left(1\right)=\cup_{m\in \mathbb{N}}H_{m}$ . Assuming *H*_*m*_ ∈ *H*° for every m∈N , this task is open.

Example 5. Never visit state ***x*** ∈ ***X*
** The fail set of this task consists of those histories that correspond to the external system arriving at *x* from some initial state *x*_0_. Since this always happens in finite time (or not at all), the fail set can be expressed as the union $\cup_{m\in \mathbb{N}}H_{m}$ , where *H*_*m*_ consists of all the histories in which state *x* is reached for the first time on stage *m*. Assuming *H*_*m*_ ∈ *H*° for all m∈N , the task is therefore closed.

Example 6. Reach state ***x*** _**1**_ while avoiding state ***x*** _**2**
_ The success set of this task may be written as the union, over m∈N , of histories which reach *x*_1_ for the first time after *m* stages, and did not visit *x*_2_ during the first *m* − 1 stages. Assuming these sets are open, the success set is thus open. With an analogous argument, it is seen that the fail set is also open.

An infinitary task is not necessarily either open or closed. One example of this are tasks that can be expressed as so-called *G*_*δ*_ sets (Dugundji, 1978, Section 3.6), that is, infinite intersections of open sets (see Example 7).

Example 7. Revisit state ***x*** ∈ ***X*** infinitely many times In this case, neither the success set or fail set of this task can be defined in terms of the sets *H*(*m*), since no finite length history can rule out either success or failure. For each m,k∈N , define $H_{m,k}=\{\bar{\eta }\in I_{hist}^{\infty }|\text{at}\;\text{stage}\;m,\text{the}\text{next}\;\text{visit}\;\text{to}\;x\;\text{happens}\;\text{after}\;k\;\text{stages\}}$ . Then, the success set is given by ${\bar{\kappa }}^{-1}\left(1\right)=\cap_{m\in \mathbb{N}}\cup_{k\in \mathbb{N}}H_{m,k}$ which is an infinite intersection of open sets if *H*_*m*,*k*_ ∈ *H*° for all *m*, *k*. This set is not generally open, but belongs to the broader class *G*_*δ*_.

In this paper, we consider tasks that are expressed as a labeling over $I_{hist}$ or those over $I_{hist}^{\infty }$ that can be transcribed as one over $I_{hist}$ . Hence, the problem families that we will introduce in Section 4.2 would refer to these types of tasks. All the tasks in examples in Section 5 are either open or closed and thus are representable by either a finitary success or failure condition. More generally, if one defines tasks using any common version of temporal logic, the corresponding success sets are always going to be Borel, that is, members of the sigma algebra generated by open sets (Dugundji, 1978, Section 3.6).

### 4.2. Problem families

It is assumed that the state-relabeled transition system (*X*, *U*, *f*, *h*, *Y*) describing the external system is fixed, but it is unknown or partially known to the observer (a robot or other observer).

Filtering (passive case) requires maintaining the label of an I-state attributed by *κ*_*task*_. Since *κ*_*task*_ is not necessarily sufficient, we cannot guarantee that the quotient system by *κ*_*task*_ is a DITS (Propositions 1 and 2). This implies that relying solely on the quotient system by *κ*_*task*_, we cannot determine the class that the current history belongs to (see the last row in (8)). Hence, we cannot determine whether a sentence describing the task is satisfied (or which sentences are satisfied).

Suppose the sets *U* and *Y* are specified, and at each stage *k*, the action *u*_*k*−1_ is known and *y*_*k*_ is observed. The following describes the problem for a passive task given a state-relabeled (history) ITS $\left(I_{hist},U\times Y,\phi_{hist},\kappa_{task},I_{task}\right)$ , in which $\kappa_{task}:I_{hist}\to I_{task}$ is a task labeling (that is not assumed to be sufficient), and $I_{task}$ is the corresponding I-space.

Problem 1. Find a sufficient I-space filter Find a sufficient refinement of *κ*_*task*_.

Note that $I_{hist}/\kappa_{task}$ determines a lower bound on the partitioning of $I_{hist}$ which is interpreted as the crucial information that cannot be relinquished without losing predictability, or success guarantees. Consequently, histories belonging to different equivalence classes with respect to *κ*_*task*_ must always be distinguished from each other.

Example 8. Goal recognition Suppose $\kappa_{task}:I_{hist}\to I_{task}$ is a labeling that partitions $I_{hist}$ into two disjoint sets; $I_{task}=\left\{\iota_{G},\iota_{NG}\right\}$ , in which ${\kappa_{task}}^{-1}\left(\iota_{G}\right)$ and ${\kappa_{task}}^{-1}\left(\iota_{NG}\right)$ correspond to histories that lead to goal and the ones that do not, respectively. Suppose the goal is recognizable, meaning that, solely based on *y*_*k*_, the value of *κ*_*task*_(*η*_*k*_) is known, for all $\eta_{k}\in I_{hist}$ and *k* > 0. Then, *κ*_*task*_ is trivially sufficient (also minimal). However, if the sensor mapping does not directly provide this information, then a refinement is needed to describe a sufficient filter that infers whether the goal is reached.

Notice that Problem 1 does not impose an upper bound. At the limit, a bijection from $I_{hist}$ is always a sufficient refinement of *κ*_*task*_. As stated previously, using history ITS can create computational obstructions in solving problems. This motivates the following problem.

Problem 2 **Find a minimal sufficient I-filter**. *Find a minimal sufficient refinement of κ*_*task*_.

We now consider a basic planning problem for which $I_{task}=\left\{0,1\right\}$ , such that ${\kappa_{task}}^{-1}\left(1\right)\subset I_{hist}$ is the set of histories that achieve the goal, and ${\kappa_{task}}^{-1}\left(0\right)\subset I_{hist}$ is its complement. Most planning problems refer to finding a labeling function *π* such that, when used to label the states of the internal system, guarantees task accomplishment. Then, *π* is called a *feasible* policy, which is formally defined in the following. Consider an external system (*X*, *U*, *f*, *h*, *Y*). Let $R_{X}\left(I_{task}\right)\subseteq X$ be the set of initial states for which there exist a k∈N and histories ${\tilde{x}}_{k}$ , ${\tilde{u}}_{k-1}$ , and ${\tilde{y}}_{k}$ , such that *x*_*i*+1_ = *f*(*x*_*i*_, *u*_*i*_) and *y*_*i*_ = *h*(*x*_*i*_) for all 0 < *i* < *k*, and $\eta_{k}\in {\kappa_{task}}^{-1}\left(1\right)$ , in which *η*_*k*_ is the history I-state corresponding to ${\tilde{u}}_{k-1}$ and ${\tilde{y}}_{k}$ . Informally, $R_{X}\left(I_{task}\right)$ is the set of initial states of the external system for which there exists an action sequence such that the evolution of the external system under this action sequence results in histories that satisfy the task description. We will then call $R_{X}\left(I_{task}\right)$ the *backward reachable set for* $I_{task}$ , analogously to the use of the same term in control theory.

Definition 6. Feasible policy for $I_{task}$
Let (I,Y,ϕ,π,U) and (*X*, *U*, *f*, *h*, *Y*) be the state-relabeled transition systems corresponding to internal and external systems, respectively. A labeling function π:I→U is a feasible policy for $I_{task}$ if for all *x* in the backward reachable set for $I_{task}$ , that is, $x\in R_{X}\left(I_{task}\right)$ , at least one history *η*_*k*_ corresponding to the coupled internal-external system (1) initialized at (*ι*_0_, *x*) belongs to ${\kappa_{task}}^{-1}\left(1\right)$ .

Most problems in the planning literature consider a fixed DITS and look for a feasible policy for $I_{task}$ . This yields the following problem. Typically, the I-space considered is *X* which makes the resulting *π* a *state-feedback policy*^6^. Note that a DITS, in other words, the robot brain, is an I-space filter itself.

Problem 3 **Find a feasible policy**. *Given* (I,Y,ϕ) *, find a labeling function* π:I→U that is *a feasible policy for* $I_{task}$ .

We can further extend the planning problem to consider an unspecified internal system. This entails finding a DITS (I,Y,ϕ) and a policy π:I→U such that the resulting histories of the coupled system $\left(I\times X,\phi \;{*}_{\pi ,h}f\right)$ belong to ${\kappa_{task}}^{-1}\left(1\right)$ , that is, they satisfy the task description. This is the problem of jointly finding an I-space-filter and a feasible policy defined over its states. Let K be the set of all I-maps defined over $I_{hist}$ . For κ∈K , let Π_*κ*_ be the set of all policies (labeling functions) that can be defined over the states of I which is the image of the mapping $\kappa :I_{hist}\to I$ .

Problem 4 **Find a DITS and a feasible policy**. *Find a pair* $\left(\kappa ,\pi \right)\in \left\{\left(\kappa ,\pi \right)|\kappa \in K\wedge \pi \in \Pi_{\kappa }\right\}$ *such that κ is sufficient and π is a feasible policy for* $I_{task}$ .

Suppose $\kappa :I_{hist}\to I$ and assume (*κ*, *π*) is a solution to Problem 4. This corresponds to the DITS $\left(I,Y,\phi_{\pi }\right)$ and a feasible policy π:I→U such that (I,Y,ϕ) is the derived ITS by *κ* and $\left(I,Y,\phi_{\pi }\right)$ is the restriction of it by *π*.

We emphasize that finding a DITS for a planning problem differs from Problems 1 and 2 in the sense that we are not looking for a refinement of *κ*_*task*_. The reason for this difference is because *κ*_*task*_ can already be sufficient; hence, it is the minimal sufficient refinement of itself. However, this does not necessarily imply the existence of a feasible policy defined over $I_{task}$ . For example, consider the *κ*_*task*_ described in Example 8 and a sensor mapping that reports whether the goal is reached or not. Even though *κ*_*task*_ is sufficient in this case, knowing when the goal is reached does not imply, in most cases, that a feasible policy exists as a labeling function for the quotient system by *κ*_*task*_, that is, over the states of $I_{task}$ . On the other hand, we can still talk about a notion of minimality. This notion is defined in the following.

Definition 7. Minimal DITS for **π** Let $\kappa :I_{hist}\to I$ and π:I→U be a solution to Problem 4. Furthermore, let $\left(I_{hist},Y,\phi_{hist,\pi \circ \kappa }\right)$ be the restriction of $\left(I_{hist},U\times Y,\phi_{hist}\right)$ by *π*∘*κ*. Denote by $\left(I\prime_{hist},Y,\phi_{hist,\pi \circ \kappa }\right)$ the subgraph of $\left(I_{hist},Y,\phi_{hist,\pi \circ \kappa }\right)$ from which the nodes that are not reachable from *η*_0_ have been pruned. We restrict the domain of I-maps *κ* and *κ*′ to $I\prime_{hist}$ . Then, (I,Y,ϕ,π,U) , determined by *κ* and *π*, is *minimal for π* if there does not exist a sufficient I-map *κ*′ with *κ* ≻ *κ*′ and a corresponding policy *π*′ for the quotient system by *κ*′ that satisfy *π*∘*κ* = *π*′∘*κ*′.

Informally, a minimal DITS for *π* implies that one cannot further reduce the quotient system by merging equivalence classes induced by *κ*, while simultaneously ensuring that when coupled to the external system that is initialized at the same state, the coupling would result in the same observation and action histories as (I,Y,ϕ,π,U) .

There may be multiple pairs of (*κ*, *π*) that solve the same problem. Given two DITS, $\left(I_{hist}/\kappa_{1},Y,\Phi_{hist}/\kappa_{1},\pi_{1},U\right)$ and $\left(I_{hist}/\kappa_{2},Y,\Phi_{hist}/\kappa_{2},\pi_{2},U\right)$ , a notion of equivalence can be determined if the preimages of *π*_1_∘*κ*_1_ and *π*_2_∘*κ*_2_ partition $I_{hist}$ in the same way. We can say that $\left(I_{hist}/\kappa_{1},Y,\phi_{hist},\pi_{1},U\right)$ requires more histories to be distinguished if the partitioning induced by *π*_1_∘*κ*_1_ is a refinement over the partitioning induced by *π*_2_∘*κ*_2_.

Suppose a feasible policy $\pi :I_{hist}\to U$ is defined over the states of the history ITS $\left(I_{hist},U\times Y,\phi_{hist}\right)$ . The restriction of the history ITS to the policy *π* is then $\left(I_{hist},Y,\phi_{hist_{\pi }}\right)$ (recall the definition given in Section 3.1 of restriction of a DITS). This is a particular case that solves Problem 4 for which (*κ*, *π*) is the pair such that $\kappa :I_{hist}\to I_{hist}$ is a bijection. Let $\left(I\prime_{hist},Y,\phi_{hist_{\pi }}\right)$ be the restriction by *π* from which the states that are not reachable from *η*_0_ are pruned. Note that $I\prime_{hist}\subseteq I_{hist}$ is the set of histories that can be realized once the history ITS is restricted by the policy *π*. Restricting the domain of *π* to $I\prime_{hist}$ , we obtain a labeling function over the states of $\left(I\prime_{hist},Y,\phi_{hist_{\pi }}\right)$ which determines the classes of histories that are distinguished by the actions selected under the policy *π*. To ensure that the same action histories are obtained when a derived DITS (quotient of the history ITS by *κ*′) is coupled to the external system, the I-map *κ*′ needs to be a refinement of *π*. Consequently, the following proposition establishes the connection between a policy *π* defined over $I_{hist}$ and its respective minimal DITS.

Proposition 3 **The minimal sufficient refinement of a feasible policy** $\pi :I_{hist}\to U$ **determines its minimal DITS**. *Let* (*κ*, *π*) *be a pair that solves Problem* 4 *such that* $\kappa :I_{hist}\to I_{hist}$ *is a bijection. Then, a minimal DITS for π is the DITS* (I,Y,ϕ) *derived from* $\left(I\prime_{hist},Y,\phi_{hist,\pi }\right)$ *by some minimal sufficient refinement κ*′ *of π*.

**Proof**. Since *κ*′ is a minimal sufficient refinement of *π*, it is sufficient and ∄*κ*″ that satisfies *κ*′⪰*κ*″⪰*π*. Since it is a refinement, every set in $I\prime_{hist}/\kappa \prime$ is a subset of $I\prime_{hist}/\pi$ . Thus, we can find a π′:I→U such that *π*′(*κ*′(*η*)) = *π*(*η*). Then, by Definition 7, (I,Y,ϕ) labeled with *π*′ is a minimal DITS for *π*.

■

### 4.3. Learning a sufficient ITS

Although learning and planning overlap significantly, some unique issues arise in pure learning (Weinstein et al., 2022). This corresponds to the case when $\kappa_{task}:I_{hist}\to I_{task}$ is not initially given but needs to be revealed through interactions with the external system, that is, respective action and observation histories. It is assumed that whether the sentence (or sentences) describing the task is satisfied or not can be assessed at a particular history I-state.

We can address both filtering and planning problems defined previously within this context, considering model-free and model-based cases. In the model-free case, the task is to compute a minimal sufficient ITS that is consistent with the actions and observations. Variations include *lifelong learning*, in which there is a single, long history I-state, or more standard learning in which the system can be restarted, resulting in multiple *trials*, each yielding a different history I-state. In the model-based case, partial specifications of *X*, *f*, and *h* may be given, and unknown parameters are estimated using the history I-state(s). Different results are generally obtained depending on what assumptions are allowed. For example, do identical history I-states imply identical state trajectories? If not, then set-based, nondeterministic models may be assumed, or even probabilistic models based on behavior observed over many trials and assumptions on probability measure, priors, statistical independence, and so on.

## 5. Applying the theory

In this section we provide some simple filtering and planning problems and show how the ideas presented in the previous sections apply to these problems. All problems defined in the previous section can be posed in a learning context as well. Then, $I_{task}$ is not given but it is revealed through interactions between the internal and external as the input-output data. Finally, we formulate as derived ITSs two established approaches, *diversity-based inference* and *predictive state representations*, for obtaining compact representations of the input-output (action-observation) relations for an unknown external system. These techniques illustrate the model-free approach to representing the internal-external coupling.

### 5.1. Red-green gates

**Figure 3.**
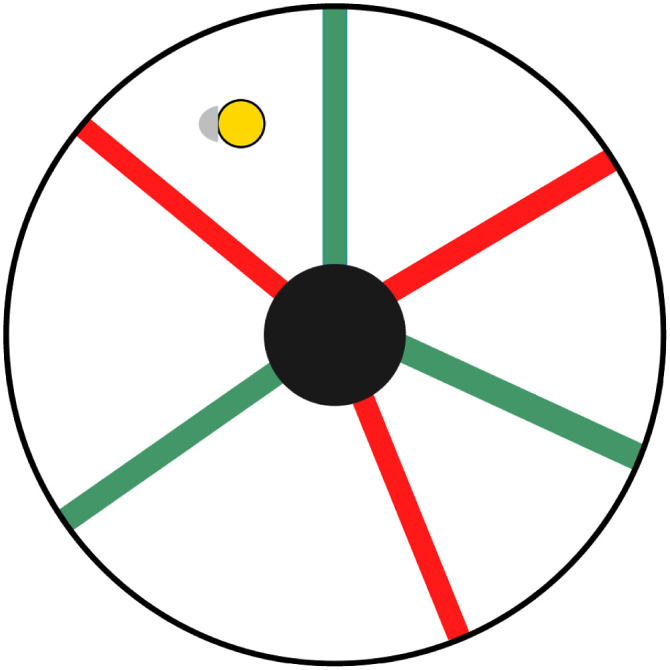
Environment used in Examples 9, 10, and the obstacle (an open disk) is shown in black.

This example is inspired by (Tovar et al., 2008). Let $E\subseteq R^{2}$ be an annulus that is partitioned into non-empty regions separated by gates, see Figure 3. Each gate is either green or red. This color can be detected by the robot’s color sensor and follows the rule that each region shares a boundary with exactly two gates; one green and one red. The set of possible observations are therefore *Y* = {*r*, *g*}. As in (Tovar et al., 2008), we assume that the robot’s trajectory is in general position with respect to the gates, in the sense that it only crosses them transversally and never goes through an intersection of two gates.

**Figure 4.**
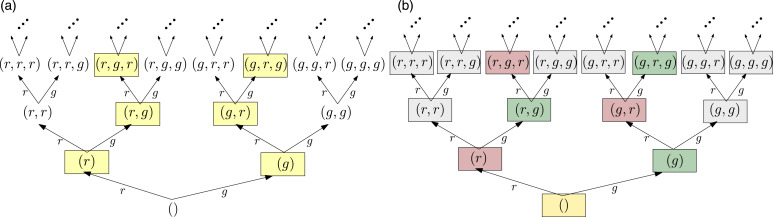
(a) State-relabeled history ITS described in Example 9, and the labeling function *κ*_*task*_. States colored yellow are the ones that do not violate the task description. (b) Equivalence classes induced by *κ*′; the minimal sufficient refinement of *κ*_*task*_.

Example 9. Consistent rotation filter This example considers a filtering problem from the perspective of an independent observer. Suppose the actions taken by the robot are not observable and the only information about the system is the history of readings coming from the robot’s color sensor; for example, (*r*, *r*, *r*, *g*, *r*, *g*). Then, the history I-space is the set of all finite length sequences of elements of *Y*, that is, $I_{hist}=Y^{*}$ , which refers to the free monoid generated by the elements of *Y* (or the Kleene star of *Y*). Hence, the history ITS can be represented as an infinite binary tree. The task is to determine whether the robot crosses the gates consistently (in a clockwise or counterclockwise manner) or not. The preimages of $\kappa_{task}:I_{hist}\to I_{task}$ partition $I_{hist}$ into two subsets: one which the condition is satisfied (so far) and the others. The labeling induced by *κ*_*task*_ is shown in Figure 4(a).

Claim 1 *Task labeling κ*_*task*_ *defined in Example* 9 *is not sufficient.*

**Proof.** There exist I-states *η*, *η*′ such that *κ*_*task*_(*η*) = *κ*_*task*_(*η*′) and there exists a *y* for which *κ*_*task*_(*ϕ*_*hist*_(*η*, *y*)) ≠ *κ*_*task*_(*ϕ*_*hist*_(*η*′, *y*)); for example consider *η* = (*r*, *g*), *η*′ = (*r*, *g*, *r*) and *y* = *g*. This shows that *κ*_*task*_ violates Definition 3.

■

**Figure 5.**
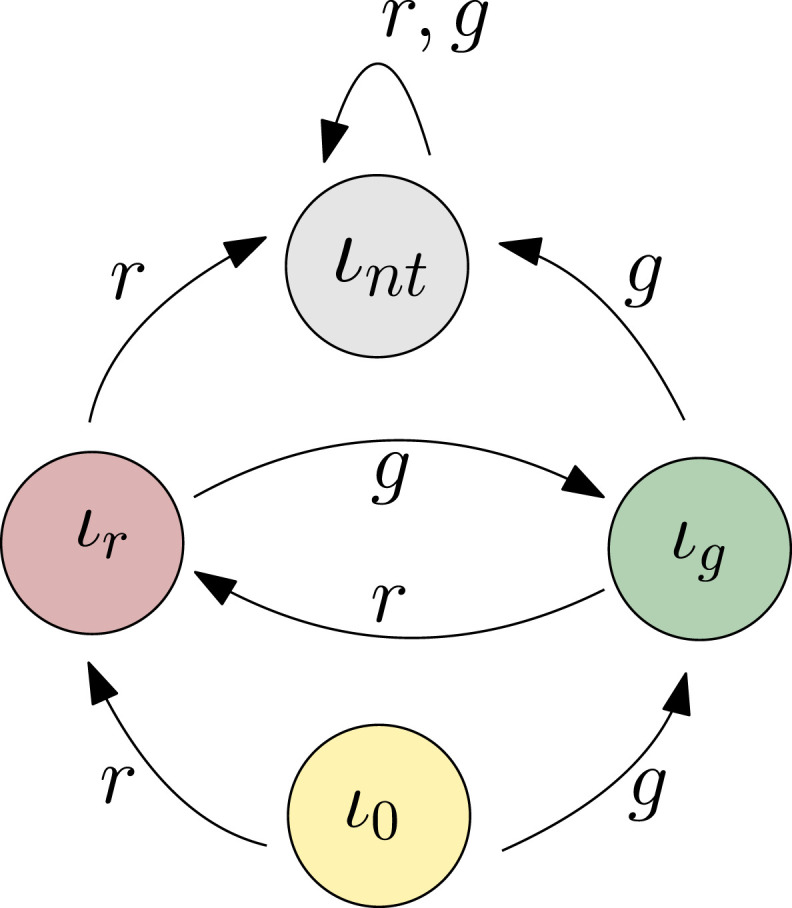
Quotient by *κ*′ of the state-relabeled history ITS shown in Figure 4(b).

We can obtain a sufficient refinement of *κ*_*task*_, defined as $\kappa \prime :I_{hist}\to \left\{\iota_{0},\iota_{r},\iota_{g},\iota_{nt}\right\}$ . The corresponding equivalence classes are shown in Figure 4(b). Its quotient DITS is shown in Figure 5.

Claim 2 *κ*′ *as defined above is a minimal sufficient refinement of κ*_*task*_.

**Proof.** It follows from Proposition 2 that if a labeling is not minimal then there is a minimal one that is strictly coarser and is still sufficient. However, neither of the subsets that belong to $I_{hist}/\kappa \prime$ can be merged, since merging *ι*_*nt*_ (colored gray in Figure 5) with anything else violates the condition that *κ*′ is a refinement of *κ*_*task*_ and any pairwise merge of the others violate sufficiency.

■

Suppose the robot has a boundary detector, and it is capable of executing a bouncing motion that involves *move forward* and *rotate in place*. The set of actions is defined as *U* = {*u*_*r*_, *u*_*g*_}, in which *u*_*g*_ represents a bouncing motion that allows the robot to traverse the green gate but not the red one, *u*_*r*_ allows it to traverse the red gate but not the green one. For all the actions, the robot also bounces off of the boundary. We assume that the boundary detector and color sensor readings do not arrive simultaneously, and that the resulting trajectory will strike every open interval in the boundary of every region infinitely often, with non-zero, non-tangential velocities (Bobadilla et al., 2011).

Example 10. Consistent rotation plan We now consider a planning problem (that belongs to the class described in Problem 4) for which the goal is to ensure that the robot crosses the gates consistently. The history I-space of the planner is $I_{hist}={\left(U\times Y\right)}^{*}$ and the preimages of *κ*_*task*_ partition $I_{hist}$ into two sets; the histories that satisfy the predicate and the ones that do not.

**Figure 6.**
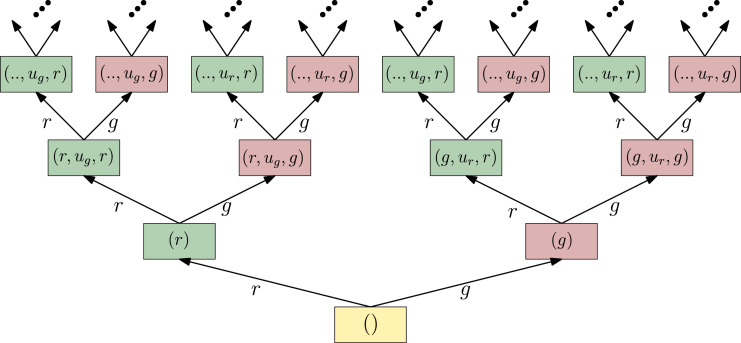
History ITS $\left(I_{hist},U\times Y,\phi_{hist}\right)$ restricted by *π*, labeled with *π*. The histories where *u*_*g*_ is applied is colored green and where *u*_*r*_ is applied in red. The initial state *η*_0_ is labeled with ().

A policy $\pi :I_{hist}\to I$ can be determined over the states of history ITS such that *π*(*η*_0_, *…*, *y*_*k*_) = *u*_*g*_ if *y*_*k*_ = *r* and *π*(*η*_0_, *…*, *y*_*k*_) = *u*_*r*_ if *y*_*k*_ = *g*. Let $\left(I\prime_{hist},Y,\phi_{hist,\pi }\right)$ be the restriction of history ITS by *π* such that the states that are not reachable from *η*_0_ are pruned (see Figure 6). The labeling *π* defined over the states of $\left(I\prime_{hist},Y,\phi_{hist,\pi }\right)$ is sufficient as can be seen from the inspection of the ITS given in Figure 6. Then, the following claim follows from Proposition 3.

Claim 3 The minimal DITS for *π* is the quotient of $\left(I\prime_{hist},Y,\phi_{hist,\pi }\right)$ by *π*.

**Figure 7.**
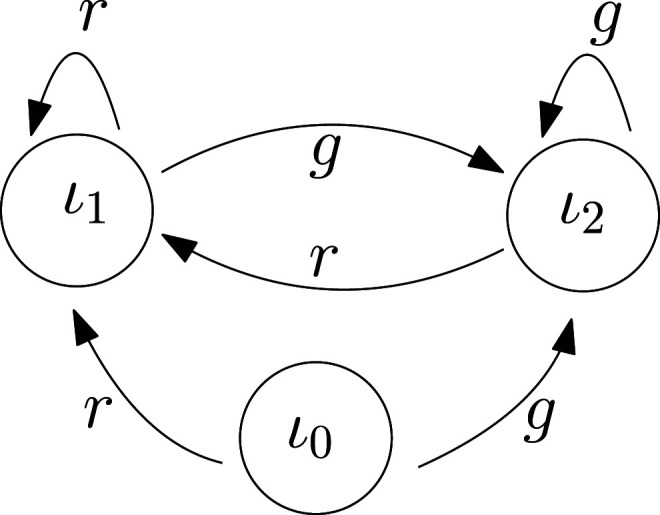
DITS describing the internal system solving the planning problem described in Example 10.

The quotient system, that is the minimal DITS, is shown in Figure 7. Let $I=\left\{i_{0},i_{1},i_{2}\right\}$ be the states of this quotient ITS. The respective plan *π*′ represented over the states of this minimal DITS is given as *π*′(*ι*_0_) = (), *π*′(*ι*_1_) = *u*_*g*_, and *π*′(*ι*_2_) = *u*_*r*_.

### 5.2. L-shaped corridor

**Figure 8.**
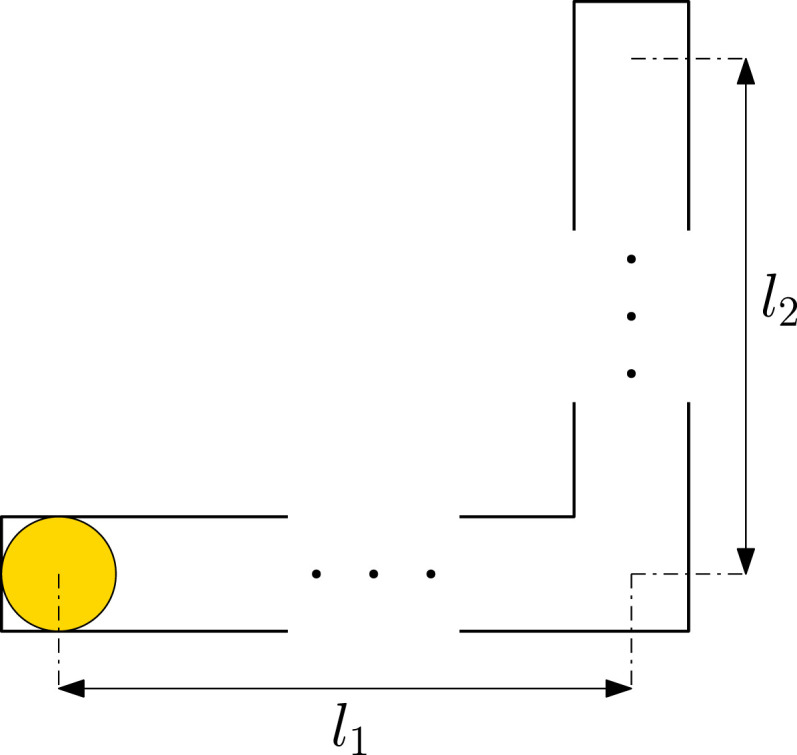
L-shaped corridor; *l*_1_, *l*_2_ ≤ *l*. For any corridor, the robot starts at the left-most part of the corridor which corresponds to the coordinates (0, 0).

Consider a robot in an inverted L-shaped planar corridor (Figure 8). Let $E_{l}$ be the set of all such environments such that *l*_1_, *l*_2_ ≤ *l*, in which *l*_1_ and *l*_2_ are the dimensions of the corridor bounded by *l*. We assume that the minimum length/width is larger than the robot radius, that is, 1. The state space *X* is defined as the set of all pairs (*q*, *E*_*i*_), in which (*q*_1_, *q*_2_) ∈ *E*_*i*_, and $E_{i}\in E_{l}$ . The action set is one which corresponds to moving one step towards right or up; if the boundary is reached, the state does not change. The robot has a sensor that reports 1 if the motion is blocked.

Example 11. L-shaped corridor Consider a model-based history ITS with *η*_0_ ⊂ *X* that specifies the initial position as *q*_0_ = (0, 0) which corresponds to the left-most bottom square of the mirrored L-shape (Figure 8) but does not specify the environment so that it can be any $E_{i}\in E_{l}$ . Let $I_{hist}$ be its set of states and let $\kappa_{ndet}:I_{hist}\to \text{pow}\left(X\right)$ be an I-map that maps the history I-state *η*_*k*_ at stage *k* to a subset of *X*_*k*_ ⊆ *X*. Since (*X*, *U*, *f*, *h*, *Y*), and *X*_0_ = *η*_0_ are known, transitions for the quotient system can be described by induction as $X_{k+1}=\hat{X}\left(X_{k},u_{k}\right)\cap H\left(y_{k+1}\right)$ , in which $\hat{X}\left(X,u\right):=\left\{f\left(x,u\right)|x\in X\right\}$ , and *H*(*y*) := *h*^−1^(*y*) ⊆ *X* is the set of all states that could yield *y*. By construction, *κ*_*ndet*_ is sufficient. Suppose $\kappa_{task}:I_{hist}/\kappa_{ndet}\to I_{task}$ is a task labeling for localization that assigns each singleton a unique label and all the other subsets are labeled the same.

Claim 4 *Let κ*_*ndet*_ *and κ*_*task*_ be the I-maps defined in Example 11*. Then, κ*_*ndet*_ is a minimal sufficient refinement of *κ*_*task*_.

**Proof.** Consider a subset *X*′ ⊆ *X* with cardinality | *X*′ | > 1 and some *x*′ ∈ *X* as labels assigned by *κ*_*ndet*_. The I-map *κ*_*task*_ is not sufficient because the transition corresponding to ( ${\left[X\prime \right]}_{\kappa_{task}},\left(u,y\right)$ ) can lead to multiple labels ${\left[x\prime \right]}_{\kappa_{task}}$ . By construction *κ*_*ndet*_ is sufficient. The I-map *κ*_*ndet*_ is a minimal sufficient refinement of *κ*_*task*_ because it is sufficient and because there does not exist a sufficient *κ* such that *κ*_*ndet*_ ≻ *κ*⪰*κ*_*task*_. Suppose to the contrary that a sufficient *κ* exists, which would mean that some equivalence classes could be merged. However, this is not possible because merging any of the non-singleton subsets violates sufficiency (as shown for *κ*_*task*_) and merging singletons with others violates that it is a refinement.

■

A policy can be described over $I_{hist}/\kappa_{ndet}$ ; *u* = (1, 0) starting from *X*_0_ until *y*_*k*_ = 1 is obtained and applying *u* = (0, 1) starting from *X*_*k*_ until *y*_*n*_ = 1 is obtained, then it is found that *q* = (*k*, *n*) and *E* is the corridor with *l*_1_ = *k*, *l*_2_ = *n*.

### 5.3. Diversity-based Inference (DBI) as a derived ITS

In this and the following section, we present DBI and its probabilistic counterpart PSR as deterministic ITSs. The core idea in DBI (Rivest and Schapire, 1993, 1994) is to gather information about the environment through action-observation experiments. The environment is modeled as a finite state Moore machine (finite state automaton), formally defined as a 6-tuple $E=\left(X,U,f,h,Y,x_{0}\right)$ . This definition coincides with our definition of the external system, but also contains an *initial state x*_0_.^7^ Experiments on E are called *tests*. Each test $t=\left({\tilde{u}}_{m},y\right)$ consists of a finite action sequence ${\tilde{u}}_{m}:=\left(u_{1},\ldots ,u_{m}\right)\in U^{m}$ , followed by an observation *y* ∈ *Y*. The test $t=\left({\tilde{u}}_{m},y\right)$ is said to *succeed* from state *x* ∈ *X*, if $\left(h\circ f^{{\tilde{u}}_{m}}\right)\left(x\right)=y$ , where

(13) $$f^{{\tilde{u}}_{m}}\left(x\right):=f\left(\cdots f\left(f\left(x,u_{1}\right),u_{2}\right)\ldots ,u_{m}\right).$$

By convention, if *m* = 0, then $f^{{\tilde{u}}_{m}}\left(x\right)=x$ . Thus, for each test *t* there exists a *success function S*_*t*_: *X* → {0, 1}, for which *S*_*t *_(*x*) = 1 if and only if *t* succeeds at *x*. In DBI, an equivalence relation ${∼}_{T}$ is defined in the set of tests $T:=\left\{\left({\tilde{u}}_{m},y\right)|{\tilde{u}}_{m}\in U^{m},y\in Y,m\in N\right\}$ by setting

$$t_{1}{∼}_{T}t_{2}\;\Leftrightarrow \;S_{t_{1}}\left(x\right)=S_{t_{2}}\left(x\right),\forall x\in X.$$

The cardinality $K:=\left|P_{T}\right|$ of the set of equivalence classes $P_{T}=\left\{\left[t\right]|t\in T\right\}$ is called the *diversity* of E . The diversity of a finite state machine satisfies *K* ≤ 2^|*X*|^ < *∞*. Each state *x* ∈ *X* can thus be labeled by a finite *success vector*

(14) $$\xi \left(x\right):=\left(S_{1}\left(x\right),\ldots ,S_{K}\left(x\right)\right)$$

in which, for *k* ∈ {1, …, *K*}, the functions $S_{k}:=S_{t_{k}}$ are the success functions of tests *t*_1_, …, *t*_*K*_, whose respective equivalence classes [*t*_1_], …, [*t*_*K*_] constitute the set $P_{T}$ .

Proposition 4 **The success vector is a minimal sufficient refinement**. *Let* $E=\left(X,U,f,h,Y,x_{0}\right)$ be a finite state Moore machine with diversity *K*. Then, *ξ*: *X* → {0,1}^*K*^ defined in (14) is a minimal sufficient refinement of *h*.

**Proof.** By setting *m* = 0 in (13), we see that *ξ* must be a refinement of *h*. Suppose *ξ*(*x*_0_) = *ξ*(*x*_1_) for some *x*_0_, *x*_1_, and let *u* ∈ *U* be arbitrary. We want to show that *ξ*(*f*(*x*_0_, *u*)) = *ξ*(*f*(*x*_1_, *u*)). Let $\left({\tilde{u}}_{m},y\right)$ be any test and let ${\tilde{v}}_{m+1}=u^{⌢}{\tilde{u}}_{m}$ denote the concatenation of *u* as a prefix to ${\tilde{u}}_{m}$ . Then,

$$\begin{array}{l} h\left(f^{{\tilde{u}}_{m}}\left(f\left(x_{0},u\right)\right)\right)=h\left(f^{{\tilde{v}}_{m+1}}\left(x_{0}\right)\right)\\ =h\left(f^{{\tilde{v}}_{m+1}}\left(x_{1}\right)\right)=h\left(f^{{\tilde{u}}_{m}}(f\left(x_{1},u\right)\right) \end{array}$$

where the middle equality follows from the assumption that *ξ*(*x*_0_) = *ξ*(*x*_1_). This means that all tests agree on *f*(*x*_0_, *u*) and *f*(*x*_1_, *u*), which implies that *ξ*(*f*(*x*_0_, *u*)) = *ξ*(*f*(*x*_1_, *u*)). Hence, *ξ* is sufficient.

Suppose *ξ* is not the minimal sufficient refinement of *h*. Let *ξ*′ be a minimal sufficient refinement of *h* which always exists, see Remark 3. Thus, we have *ξ* ≻ *ξ*′⪰*h*, so there are *x*_0_, *x*_1_ ∈ *X* with

(15) $$\left(i\right)\xi^{\prime }\left(x_{0}\right)=\xi^{\prime }\left(x_{1}\right)\;\;\;\;\text{and }\;\left(\text{ii}\right)\xi \left(x_{0}\right)\neq \xi \left(x_{1}\right).$$

Since *ξ*′ is sufficient, it follows from (15) (i) that *ξ*′(*f*(*x*_0_, *u*)) = *ξ*′(*f*(*x*_1_, *u*)) for all actions *u* ∈ *U*. Using the sufficiency of *ξ*′ again *m* times, it follows that

(16) $$\xi^{\prime }\left(f^{{\tilde{u}}_{m}}\left(x_{0}\right)\right)=\xi^{\prime }\left(f^{{\tilde{u}}_{m}}\left(x_{1}\right)\right)$$

for all finite action sequences ${\tilde{u}}_{m}\in U^{m}$ . Now, since *ξ*′ is a refinement of *h*, (16) implies that $h\left(f^{{\tilde{u}}_{m}}\left(x_{0}\right)\right)=h\left(f^{{\tilde{u}}_{m}}\left(x_{1}\right)\right)$ for all ${\tilde{u}}_{m}\in U^{m}$ . By definition, this means that any test $\left({\tilde{u}}_{m},y\right)$ succeeds from *x*_0_ if and only if it succeeds from *x*_1_. This implies *ξ*(*x*_0_) = *ξ*(*x*_1_), contradicting (15) (ii), and proves the claim.

■

Now, denote the concatenation of an action *u*_0_ ∈ *U* and a test $t=\left({\tilde{u}}_{m},y\right)\in U^{m}\times Y$ by

$${u_{0}}^{⌢}t:=\left(u_{0},u_{1},\ldots ,u_{m},y\right)\in U^{m+1}\times Y.$$

Since $S_{t}\left(f\left(x,u\right)\right)=S_{u^{⌢}t}\left(x\right)$ for all t∈T and *u* ∈ *U*, there exists for each *u* ∈ *U* a well-defined mapping $g_{u}:P_{T}\to P_{T}$ given by $g_{u}\left(\left[t\right]\right):=\left[u^{⌢}t\right]$ . Furthermore, each *g*_*u*_ defines a mapping (not necessarily a permutation) *α*_*u*_: {1, …, *K*} → {1, …, *K*} by

(17) $$\alpha_{u}\left(k\right)=n\Leftrightarrow g_{u}\left(\left[t_{k}\right]\right)=\left[t_{n}\right].$$

Definition 8. Update graph Let $E=\left(X,U,f,h,Y,x_{0}\right)$ be a finite state Moore machine with diversity $K=\left|P_{T}\right|$ . Let $P_{T}=\left\{\left[t_{1}\right],\ldots ,\left[t_{K}\right]\right\}$ be the set of test equivalence classes with representatives $t_{1},\ldots ,t_{K}\in T$ , and let *S*_*k*_ be the success function of test *t*_*k*_. Finally, let *α*_*u*_ be as in (17). The *update graph* of E is a state-relabeled deterministic transition system $G:=\left(S,U,\tau ,\sigma ,Y,s_{0}\right)$ where *U* and *Y* are as in E , and

 • $S:=\left\{\left(S_{1}\left(x\right),\ldots ,S_{K}\left(x\right)\right)\in {\left\{0,1\right\}}^{K}|x\in X\right\},$
 • $\tau \left(\left(s_{1},\ldots ,s_{K}\right),u\right):=\left(s_{\alpha_{u}\left(1\right)},\ldots ,s_{\alpha_{u}\left(K\right)}\right),$
 • $\sigma \left(\left(s_{1},\ldots ,s_{K}\right)\right)=h\left(x\right)$ , where *x* ∈ *X* is such that *s*_*k*_ = *S*_*k*_(*x*) for all *k* ∈ {1, …, *K*}, and
 • *s*_0_ = (*S*_1_(*x*_0_), *…*, *S*_*K*_(*x*_0_)).

A machine/environment E is said to be *reduced*, if for each state *x* ∈ *X* there exist tests $t_{1},t_{2}\in T$ for which $S_{t_{1}}\left(x\right)\neq S_{t_{2}}\left(x\right)$ . It was shown in (Rivest and Schapire, 1993, Theorem 3) that it is possible to simulate a reduced environment E by its update graph. We rephrase and prove this result in terms of isomorphisms of transition systems. Two Moore machines (*X*, *U*, *f*, *h*, *Y*, *x*_0_) and $\left(X\prime ,U,f\prime ,h\prime ,Y,x\prime_{0}\right)$ (both defined in terms of the same action and observation sets *U* and *Y*) are said to be *isomorphic*, if there exists a bijective map *g*: *X* → *X*′ such that for all *x* ∈ *X* and *u* ∈ *U* we have f′(g(x),u)=g(f(x,u)) , $g\left(x_{0}\right)=x\prime_{0}$ , and (*h*′∘*g*) (*x*) = *h*(*x*). The following is essentially (Rivest and Schapire, 1993, Theorem 3):

Proposition 5 **Update graph representation of a Moore machine**. *Let* $E=\left(X,U,f,h,Y,x_{0}\right)$ *be a finite state Moore machine with a reduced state space X and let* $G:=\left(S,U,\tau ,\sigma ,Y,s_{0}\right)$ *be the update graph of* E . *Then, the function ξ from* (14) *is an isomorphism between* E *and* G .

**Proof.** Let $P_{T}=\left\{\left[t_{1}\right],\ldots ,\left[t_{K}\right]\right\}$ be the set of test equivalence classes in E with representatives $t_{1},\ldots ,t_{K}\in T$ , and let *S*_*k*_ be the success function of test *t*_*k*_. Recall the definition of *ξ* from (14).

Then, *ξ*: *X* → *S* is onto by definition of the set *S*. To show injectivity, assume *ξ*(*x*) = *ξ*(*x*′) for some *x*, *x*′ ∈ *X*, which means that *S*_*k*_(*x*) = *S*_*k*_(*x*′) for all *k* = 1, …, *K*. Since E is reduced, it suffices to show that *S*_*t*_(*x*) = *S*_*t*_(*x*′) for all tests t∈T because then *x* = *x*′. Since every test *t* satisfies [*t*] = [*t*_*k*_] for some *k*, the claim follows immediately.

We still need to show that for all (*x*, *u*) ∈ *X* × *U*, the functions *f*_*u*_(*x*):= *f*(*x*, *u*) and *τ*_*u*_(*s*):= *τ*(*s*, *u*) satisfy $\left(\tau_{u}\circ \xi \right)\left(x\right)=\left(\xi \circ f_{u}\right)\left(x\right)$ . According to Definition 8, each *x* ∈ *X* and *u* ∈ *U* satisfies $\left(\tau_{u}\circ \xi \right)\left(x\right)=\left(S_{\alpha_{u}\left(1\right)}\left(x\right),\ldots ,S_{\alpha_{u}\left(K\right)}\left(x\right)\right)$ where *α*_*u*_(*k*) = *n* iff [*t*_*n*_] = [*u*^⌢^*t*_*k*_]. Thus,

(18) $$\begin{array}{l} \left(\tau_{u}\circ \xi \right)\left(x\right)=\left(S_{\alpha_{u}\left(1\right)}\left(x\right),\ldots ,S_{\alpha_{u}\left(K\right)}\left(x\right)\right)\\ =\left(S_{u^{⌢}t_{1}}\left(x\right),\ldots ,S_{u^{⌢}t_{K}}\left(x\right)\right)\\ =\left(S_{1}\left(f\left(x,u\right)\right),\ldots ,S_{K}\left(f\left(x,u\right)\right)\right)\\ =\xi \left(f\left(x,u\right)\right)=\left(\xi \circ f_{u}\right)\left(x\right). \end{array}$$

Since *ξ* is a bijection, the labeling function *σ* in Definition 8 is well defined, and satisfies (*σ*∘*ξ*) (*x*) = *h*(*x*) by definition. Finally, *ξ*(*x*_0_) = *s*_0_ by the definition of *s*_0_.

■

To simulate the environment E by G , one needs to set the initial state ${\hat{s}}_{0}=\left(s_{1},\ldots ,s_{K}\right)\in S$ , which corresponds to the initial state *x*_0_ ∈ *X* for which *S*_*k*_(*x*_0_) = *s*_*k*_ for all *k* ∈ {1, …, *K*}.

If we remove the assumption that E is reduced, we can view the function *ξ*: *X* → *S* in (14) as a labeling that identifies those pairs of states that cannot be differentiated by any test.

Proposition 6 **Update graph representation is a DITS**. *Let* $T_{E}=\left(X,U,f,h,Y\right)$ *be the transition system corresponding to the finite state Moore machine* $E=\left(X,U,f,h,Y,x_{0}\right)$ *, and let* G:=(S,U,τ,σ,Y) *be the update graph of* E . *Then,* G *is a DITS.*

**Proof.** According to Proposition 4, *ξ*: *X* → *S* in (14) is a sufficient labeling for $T_{E}$ . By definition, any two states (equivalence classes) [*x*_1_], [*x*_2_] of the quotient system $T_{E}/\xi$ satisfy [*x*_1_] ≠ [*x*_2_] if and only if *ξ*(*x*_1_) ≠ *ξ*(*x*_2_). This implies that $T_{E}/\xi$ defines a reduced Moore machine which is isomorphic to G according to Proposition 5. The claim then follows from Remark 1, which states that quotients by sufficient labelings are deterministic transition systems.

■

### 5.4. Predictive state representations (PSRs)

*Predictive state representation (PSR)* (James and Singh, 2004; Littman and Sutton, 2001), like its deterministic predecessor DBI, is based on the idea of performing tests on the environment. The difference is that PSR assumes a statistical description of the internal-external coupling, expressed via success probabilities of tests, conditioned on past histories. Predictive state representations have been shown to be more general than POMDPs (Cassandra et al., 1997) in the sense that every POMDP model can be represented via the corresponding PSR.

Since the introduction of the original PSR, several variations of the concept have been proposed in connection to different learning algorithms that aim to discover the set of core tests and learn the associated prediction functions (Boots et al., 2011, 2013; James and Singh, 2004). So-called TPSRs (Rosencrantz et al., 2004) are adaptations of the concept where, instead of maintaining vectors of probabilities over a finite set of core tests, a linear combination of a larger set of tests is maintained instead.

We focus on the original formulation of the PSR model and show how it can be expressed in our formalism as a DITS. Let $\eta_{k}:=\left({\tilde{u}}_{k-1},{\tilde{y}}_{k}\right)$ denote the *history I-state* at stage *k* (including the *k*th observation, but not the *k*th action). In addition, let ${\tilde{y}}_{k,m}:=\left(y_{k},\ldots ,y_{k+m}\right)$ and similarly ${\tilde{u}}_{k,m}:=\left(u_{k},\ldots ,u_{k+m}\right)$ for all m∈N . In PSR, action-observation sequences $t=\left({\tilde{u}}_{m-1}^{t},{\tilde{y}}_{m}^{t}\right)$ are called *tests*. At stage k∈N , a PSR model maintains a sufficient statistic for computing the conditional *success probabilities*

(19) $$P_{\eta_{k}}\left(t\right):=P\left({\tilde{y}}_{k,m}={\tilde{y}}_{m}^{t}|{\tilde{u}}_{k,m-1}={\tilde{u}}_{m-1}^{t},\eta_{k}\right)$$

for tests $t=\left({\tilde{u}}_{m-1}^{t},{\tilde{y}}_{m}^{t}\right)$ of arbitrary length *m* ≥ 1.

The idea of PSR is to identify minimal *core sets of tests Q* = (*t*_1_, …, *t*_*m*_) which have the property that, given any test *t*∉*Q* and some history $\eta_{k}\in I_{hist}$ , it is possible to compute the success probability of *t* as $P_{\eta_{k}}\left(t\right)=f_{t}\left(Q\left(\eta_{k}\right)\right)$ where $Q\left(\eta_{k}\right):=\left(P_{\eta_{k}}\left(t_{1}\right),\ldots ,P_{\eta_{k}}\left(t_{m}\right)\right)$ is the *prediction vector* for the set *Q* and *f*_*t*_ is the *prediction function* associated with *t*. In linear PSR, the space of admissible prediction functions is restricted to linear transformations (vectors) $r_{t}\in R^{\left|Q\right|}$ so that *f*_*t*_(*Q*(*η*_*k*_)) = *r*_*t*_ ⋅ *Q*(*η*_*k*_) for all *t*, *η*_*k*_.

Formally, a PSR is a 5-tuple (*U*, *Y*, *Q*, *F*, *m*_0_), where *U* is the set of actions, *Y* is the set of observations, *Q* is a *core set of tests*, *F* is the set of *prediction functions*, and *m*_0_ ∈ [0,1]^|*Q*|^ is the *initial prediction vector* after seeing the null history *η*_0_ = (). A PSR model provides a complete (probabilistic) description of the action-observation dynamics because the prediction vector *Q*(*η*_*k*_) can be updated with each new action-observation. For this, only the (finite number of) prediction functions *f*_(*u*,*y*)_ and $f_{{\left(u,y\right)}^{⌢}t_{m}}$ need to be known, corresponding to all possible action-observation pairs (*u*, *y*) and to concatenations of these with the core tests *t*_*m*_ ∈ *Q*. Then, the update to *Q*(*η*_*k*+1_), where *η*_*k*+1_ = *η*_*k*_^⌢^(*u*, *y*), is obtained through the function *ϕ*_PSR_: [0,1]^*m*^ × (*U* × *Y*) → [0,1]^*m*^ defined by

(20) $$\begin{array}{l} \phi_{\text{PSR}}\left(Q\left(\eta_{k}\right),\left(u,y\right)\right):=\\ \;\;\;\;\;\;\left(\phi_{1}\left(Q\left(\eta_{k}\right),\left(u,y\right)\right),\ldots ,\phi_{m}\left(Q\left(\eta_{k}\right),\left(u,y\right)\right)\right), \end{array}$$

where the functions *ϕ*_*i*_: [0,1]^*m*^ × (*U* × *Y*) → [0, 1] are given for each *i* ∈ {1, …, *m*} by

$$\begin{array}{l} \phi_{i}\left(Q\left(\eta_{k}\right),\left(u,y\right)\right):=P_{{\eta_{k}}^{⌢}\left(u,y\right)}\left(t_{i}\right)\\ =\frac{P_{\eta_{k}}\left({\left(u,y\right)}^{⌢}t_{i}\right)}{P_{\eta_{k}}\left(\left(u,y\right)\right)}=\frac{f_{{\left(u,y\right)}^{⌢}t_{i}}\left(Q\left(\eta_{k}\right)\right)}{f_{\left(u,y\right)}\left(Q\left(\eta_{k}\right)\right)}. \end{array}$$

Thus, a PSR with a core set of tests *Q* = (*t*_1_, …, *t*_*m*_) is a DITS $\left(I_{\text{PSR}},U\times Y,\phi_{\text{PSR}}\right)$ , where $I_{\text{PSR}}:=\left\{Q\left(\eta_{k}\right)|\eta_{k}\in I_{hist}\right\}$ . The corresponding I-map $\kappa_{\text{PSR}}:I_{hist}\to I_{\text{PSR}}$ is given by *κ*_PSR_(*η*):= *Q*(*η*).

## 6. Conclusions and future work

This paper introduced a mathematical framework for determining minimal filters and minimal feasible policies by comparing ITSs over information spaces. The minimality results are quite general without imposing strong restrictions on the underlying dynamical system (external system). We show that a large class of problems can be posed and analyzed under this framework.

Nevertheless, there are several opportunities to expand the general theory. For example, we assumed that *u* is both the output of a policy and the actuation stimulus in the physical world; more generally, we should introduce a mapping from an action symbol *σ* ∈ Σ to a control function $\tilde{u}\in \tilde{U}$ so that plans are expressed as π:I→Σ and each *σ* = *π*(*ι*) produces energy in the physical world via a mapping from Σ to $\tilde{U}$ .

It is also important to extend the models to continuous time. In this case, the sensing and action histories are time parameterized functions, rather than sequences. Sufficiency must be defined in terms information mappings that apply to any time slice from 0 to *t*′ < *t* for a history that runs from time 0 to *t*, rather than only over discrete time steps. Some ground work has already been done in (LaValle, 2006).

Another direction is to consider the hardware and actuation models as variables, and fix other model components. This is similar to the class of problems related to co-design for which the design process of a robot given resource constraints (sensors and actuators) is sought to be automated (Shell et al., 2021; Censi, 2016; Zardini et al., 2021).

In this paper, we considered the theoretical limits on the DITS necessary to express a policy defined over a history ITS. However, the problem of finding such a DITS remains as an open algorithmic challenge. Furthermore, we only considered feasible policies. An interesting direction is to analyze the information requirements for policies that are optimal with respect to a relevant objective and the trade-off between optimality and minimality. This will amount to an ordering (potentially a partial ordering) of policies in terms of (expected) cost and the minimal DITS to express such policy.

In an external-internal coupled system, the different components, I-map *κ*, the information transition function *ϕ* and the policy *π* share the total complexity (information content) of the internal information processing system. Of particular interest would be to explore the trade-offs between these components in terms of efficient encoding of data-structures and their successful decoding in terms of policies. Ultimately this could lead to fundamental characterizations of interaction system information content in the spirit of the minimum description length principle proposed in (Rissanen, 1978).

The mathematical theory of coupling as presented in this paper is very general. Coupling in discrete dynamical systems, and of finite automata, are special cases of it, and even continuous systems can be seen as such. Connections to other work on coupling such as (Spivak, 2015) are to be explored. Dynamic coupling has been proposed as a viable approach to a mathematical modeling of cognition from the enactivist perspective (Montebelli et al., 2008; Favela, 2020). The existing literature on the latter uses bits and pieces of dynamical systems with sporadic applications in different areas of cognitive science, but a systematic unifying study is still to be seen, especially one that has meaningful ramifications to robotics and algorithmic design. An attempt to connect philosophical ideas with those of this paper was presented by the authors in (Weinstein et al., 2022).

A grand challenge remains: The results here are only a first step toward producing a more complete and unique theory of robotics that clearly characterizes the relationships between common tasks, robot systems, environments, and algorithms that perform filtering, planning, or learning. We should search for lattice structures that play a role similar to that of language class hierarchies in the theory of computation. This includes the structures of the current paper and the sensor lattices of (LaValle, 2012; Zhang and Shell, 2021). Many existing filtering, planning, and learning methods can be formally characterized within this framework, which would provide insights into relative complexity, completeness, minimality, and time/space/energy tradeoffs.

## Notes

1. In dynamical systems terminology, the coupled system is a closed, deterministic dynamical system. In the presence of extrinsic disturbances (see Section 2.2), the coupled system becomes a non-autonomous dynamical system.

2. Although the values of the disturbances, when present, are not known beforehand or deterministically, once the disturbed parts of the coupled system receive the value of the disturbance, they produce a uniquely determined output. Hence, these components and their couplings are deterministic transition systems.

3. Considering homomorphisms between transition systems goes beyond the present paper; a good source for the general theory is (Goranko and Otto, 2007).

4. Note that we can treat the external system symmetrically and given a nondeterministic transition system (*X*, *Y* × *U*, *F*) with *F* ⊆ *X* × (*Y* × *U*) × *X* and a sensor mapping *h*: *X* → *Y*, we can define the restriction *F*_*h*_ in an analogous way as above. However, because it is not central to this paper we will not elaborate on this topic.

5. A transition system (*S*, Λ, *T*) is full, if *∀s* ∈ *S*, *λ* ∈ Λ there exists at least one *s*′ ∈ *S* with (*s*, *λ*, *s*′) ∈ *T*.

6. The term *state* in state-feedback policy refers to the states of the external system, as it is commonly used in the robotics literature.

7. In (Rivest and Schapire, 1993), the authors allow several different observations (predicate symbols) *p* ∈ *P* to be observed at each state via a function *γ*: *X* × *P* → {0, 1}. We assume for simplicity that each *x* ∈ *X* corresponds to a unique observation *y* = *h*(*x*).
